# Gut Microbiome–Hormone Interactions and Precision Fermentation in the Prevention of Early Cardiovascular Risk in Adolescents

**DOI:** 10.3390/ijms27125309

**Published:** 2026-06-11

**Authors:** Natalia Kurhaluk, Anna Rymuszka, Renata Kołodziejska, Zbigniew Mazur, Halina Tkaczenko

**Affiliations:** 1 Institute of Biology, Pomeranian University in Słupsk, Arciszewski St. 22B, 76-200 Słupsk, Poland; zbigniewmazur1@wp.pl; 2 Department of Physiology and Toxicology, Faculty of Medicine, The John Paul II Catholic University of Lublin, 1I Konstantynów St., 20-708 Lublin, Poland; anna.rymuszka@kul.pl; 3 Department of Medical Biology and Biochemistry, Collegium Medicum in Bydgoszcz, Nicolaus Copernicus University in Toruń, M. Karłowicz St. 24, 85-092 Bydgoszcz, Poland; renatak@cm.umk.pl

**Keywords:** adolescence, cardiovascular risk, gut microbiome, diet–microbiome–hormone axis, precision fermentation, Poland

## Abstract

Adolescence is a developmental stage marked by dynamic interactions between diet, the gut microbiome and endocrine maturation, creating a physiological environment in which early metabolic disturbances can rapidly translate into long-term cardiovascular vulnerability. This narrative review summarises the latest research on the diet–microbiome–hormone axis in adolescents, focusing on the metabolic pathways through which microbial metabolites influence host physiology. Short-chain fatty acids (SCFAs), microbially transformed bile acids and postbiotic signalling molecules regulate enteroendocrine communication, insulin sensitivity, vascular function and inflammatory tone, thereby linking dietary exposures to early cardiometabolic alterations. Dysbiosis, driven by ultra-processed dietary patterns, low fibre intake and reduced microbial diversity, promotes metabolic endotoxemia, neuroendocrine imbalance and endothelial impairment, all of which are recognised as early indicators of cardiovascular disease. A distinctive contribution of this review is the integration of PF into the adolescent cardiometabolic framework. This emerging biotechnological process enables the controlled production of structurally defined bioactive compounds, including angiotensin-converting enzyme (ACE) inhibitory peptides, targeted prebiotic oligosaccharides, fermentable substrates that promote SCFA formation, microbially derived eicosapentaenoic acid (EPA) and docosahexaenoic acid (DHA), phytosterols and purified postbiotics. These compounds modulate several regulatory pathways, such as the renin–angiotensin–aldosterone system, lipid and bile acid metabolism, gut barrier stability, inflammatory signalling and endocrine axes involving glucagon-like peptide-1 (GLP-1), peptide YY (PYY), leptin, insulin sensitivity and growth hormone/insulin-like growth factor-1 (GH/IGF-1) dynamics. By situating precision fermentation within the broader context of adolescent metabolic susceptibility, this review highlights its potential to support microbiome resilience, stabilise hormonal regulation and mitigate early cardiovascular risk. However, further adolescent-specific clinical trials and long-term safety assessments are required to translate these advances into effective public health strategies.

## 1. Introduction

Adolescence, defined as the developmental period between 10 and 19 years of age, represents a critical window of physiological, hormonal and metabolic transformation during which rapid changes in body composition, endocrine function and metabolic regulation heighten sensitivity to environmental influences such as diet and lifestyle, while accelerated growth, pubertal hormonal activation and the maturation of metabolic regulatory pathways markedly increase nutritional demands and overall metabolic plasticity [1]. There is evidence to suggest that unhealthy dietary habits and behaviours established during this period can have long-lasting consequences, increasing the risk of being overweight or obese, as well as the development of early markers of cardiovascular disease (CVD) in adulthood [1,2]. These early exposures shape metabolic trajectories that persist into adulthood, highlighting adolescence as a critical factor in determining long-term cardiometabolic health.

This period is also crucial for the maturation of energy metabolism and hormonal regulation. The pubertal transition involves complex interactions within the endocrine system, including the hormones growth hormone (GH), insulin-like growth factor-1 (IGF-1), sex steroids, leptin and insulin. These hormones play a central role in regulating energy balance, body composition and metabolic efficiency [3]. However, inadequate dietary intake, chronic low-grade inflammation, or oxidative stress can disrupt hormonal balance, impacting insulin, IGF-1, leptin, and sex steroid signalling. These disturbances can directly contribute to the development of insulin resistance, dyslipidaemia and hypertension, which are key determinants of early cardiovascular risk [4]. Longitudinal cohort studies consistently demonstrate that early dietary patterns and physical activity levels are strongly associated with metabolic risk profiles in adulthood [5]. Emerging evidence also suggests that modulation of endocrine pathways by diet may be mediated by microbial metabolites produced in the gastrointestinal tract. This highlights the growing importance of the diet–microbiome–hormone axis in programming cardiometabolic health [6,7].

In recent years, the role of the gut microbiome in shaping cardiometabolic health during adolescence has received increasing attention. The composition and function of the gut microbiota are highly responsive to dietary inputs and influence immune regulation, lipid metabolism, glucose homeostasis, and bile acid signalling, all of which are central to cardiovascular physiology [8]. Dysbiosis, or disruption of the microbial balance, can lead to increased production of pro-inflammatory metabolites such as trimethylamine N-oxide (TMAO) and reduced synthesis of beneficial SCFAs. These changes may promote chronic inflammation and endothelial dysfunction [9,10]. As the gut microbiome continues to develop during adolescence, dietary patterns, lifestyle behaviours and environmental exposures shape its maturation and long-term metabolic programming [11]. SCFAs, including acetate, propionate and butyrate, influence host endocrine activity, appetite regulation and lipid metabolism, thereby linking microbial fermentation processes to systemic hormonal pathways and early cardiovascular risk mechanisms [12,13].

Concurrently, the increasing consumption of ultra-processed foods and the growing prevalence of sedentary lifestyles among adolescents are recognised as key factors contributing to rising rates of obesity, hypertension, and metabolic disturbances in young populations. These trends translate into an elevated cardiovascular risk that may manifest early in adulthood [14,15]. Studies show that poor dietary quality during adolescence, including low intake of fruits and vegetables and high consumption of saturated fats, is associated with adverse lipid profiles and other markers of CVD risk [16,17]. Furthermore, the literature emphasises that sedentary behaviour, sleep disturbances, and inadequate dietary habits are all independently associated with increased cardiometabolic risk later in life [18]. Clinical guidelines and scientific reviews increasingly highlight the need for early preventive interventions, such as nutrition education, promotion of physical activity, and lifestyle modification, to mitigate the growing burden of cardiovascular disease [19]. In this context, innovative food technologies, including precision fermentation (PF), are being explored as sustainable strategies for producing functional food ingredients that may modulate gut microbiota composition, improve metabolic homeostasis, and support preventive nutritional approaches aimed at reducing early cardiometabolic risk [20].

The gut microbiome plays a central role in regulating host metabolic pathways, including those related to energy homeostasis, glucose and lipid metabolism, and bile acid turnover [21]. Gut microorganisms regulate host metabolic signalling, immune responses and intestinal barrier integrity through the production of microbial metabolites such as short-chain fatty acids (SCFAs), secondary bile acids and indole derivatives. These metabolites also affect psychological well-being and neuroendocrine function [22]. Increasing evidence shows that the gut microbiota communicates with the endocrine system via the gut–brain and gut–liver axes, shaping appetite control, stress-related responses and hormonal signalling pathways [23]. Together, these interconnected diet–microbiome–hormone interactions provide a conceptual basis for understanding how cardiometabolic risk factors emerge during adolescence [1].

Building on this concept, PF represents a link between the biological vulnerabilities of adolescence and the need for tailored nutritional strategies capable of modulating the diet–microbiome–hormone axis involved in early cardiovascular risk development. During this period, metabolic pathways, endocrine signalling, and gut microbiome composition are still maturing, making adolescents particularly responsive to bioactive dietary components [24,25]. PF enables the production of highly specific functional ingredients, such as postbiotics, tailored oligosaccharides, bioactive peptides, microbial metabolites, and enzyme-modulating compounds, which can interact directly with these developing systems. Unlike traditional fermentation, PF uses genetically characterised microorganisms and controlled bioprocesses to synthesise defined molecules with targeted biological activity [26,27]. As these ingredients can be engineered to influence microbial ecology, inflammatory signalling, lipid and glucose metabolism, and hormone receptor pathways, they offer a level of precision that cannot be achieved through conventional foods or supplements.

This is particularly relevant in the context of dysbiosis, oxidative stress, and hormonal imbalances observed in adolescents exposed to ultra-processed diets, sedentary lifestyles, and metabolic stressors. These factors may contribute to the premature development of cardiovascular disease [1,5,10,28]. Adolescents consuming diets high in refined sugars, saturated fats, and ultra-processed foods often exhibit reduced gut microbial diversity and decreased production of protective metabolites such as SCFAs, which may exacerbate systemic inflammation and endothelial dysfunction [29]. Studies suggest that PF can produce compounds that enhance SCFA production, reduce TMAO-related pathways, stabilise insulin and IGF-1 signalling, and support microbial diversity. This makes it a sustainable and scientifically grounded approach to mitigating early cardiometabolic risk [30]. Furthermore, fermentation-derived ingredients can modulate host-microbe interactions, thereby influencing metabolic signalling pathways involved in lipid metabolism, insulin sensitivity, and inflammatory regulation [31]. The reliance of this process on controlled microbial biosynthesis ensures reproducibility, purity, and environmental efficiency, aligning with global sustainability goals while addressing the specific physiological needs of adolescents [10].

Although adolescence is increasingly recognised as a critical period for shaping long-term cardiometabolic health, the literature on how emerging food technologies, particularly PF, can provide functional ingredients to modulate diet–microbiome–hormone interactions remains limited. PF allows for the production of highly specific bioactive compounds, including postbiotics, peptides, oligosaccharides and microbial metabolites. These compounds have the potential to influence metabolic signalling, gut microbial ecology and inflammatory pathways that are relevant to the early development of CVD [32,33].

While many studies have examined the effects of diet or microbiota in isolation, analyses that comprehensively integrate nutritional interventions, microbial metabolism, and endocrine regulation during adolescence are scarce. To date, no review has addressed both adolescent metabolic vulnerabilities and the potential of precision-fermented functional ingredients to modulate these pathways in a sustainable and clinically meaningful manner. This article aims to address this gap by synthesising biochemical, nutritional, microbiological and clinical evidence in order to evaluate the potential of PF as a strategy for reducing early cardiovascular risk. To ensure conceptual clarity, this review first outlines the diet–microbiome–hormone system that shapes early cardiovascular vulnerability in adolescents. It then discusses PF as a forward-looking nutritional strategy that builds upon these established pathways.

The aim of this review is to characterise the physiological, hormonal, and microbiome-related determinants of early cardiovascular risk in adolescents, and to summarise current evidence on diet–microbiome–hormone interactions relevant to cardiometabolic health. It also describes the technological principles and sustainability advantages of PF and evaluates bioactive compounds produced through this process that may modulate metabolic, microbial, and hormonal pathways in adolescents. Finally, it identifies research gaps and future directions for integrating these ingredients into adolescent nutrition strategies. In light of the increasing cardiometabolic risk among adolescents in Central and Eastern Europe, this review incorporates data from the Polish population to provide a region-specific epidemiological context that supplements the broader biological and nutritional framework. Particular emphasis is placed on the role of sustainable biotechnology in developing next-generation functional foods that support the prevention of cardiometabolic disease during critical developmental periods. Where available, data from the Polish population are also incorporated to enhance contextual relevance.

## 2. Literature Search and Study Selection

A structured literature search was conducted across several major scientific databases, including PubMed, Scopus, Web of Science, Embase and Google Scholar. Publications up to the year 2025 were included. The search strategy employed a variety of keywords, including “adolescence”, “cardiovascular risk”, “microbiome”, “hormonal regulation”, “precision fermentation”, “functional ingredients”, “postbiotics”, “bioactive compounds”, “Poland”, and “sustainable nutrition”. Studies were included if they were peer-reviewed original research, reviews or meta-analyses; involved adolescents aged 10–19.9 years or biological pathways relevant to this age group; focused on diet–microbiome–hormone interactions or examined PF or fermentation-derived functional ingredients; and were published in English. Exclusion criteria included non-scientific reports, commentaries or opinion pieces without empirical data; studies unrelated to cardiometabolic or microbiome-related outcomes; research focused solely on adults with no relevance to adolescence; and articles lacking methodological transparency. Where direct evidence in adolescents was limited, studies conducted in adults or experimental models were included if their findings were relevant to pathways involved in adolescent metabolic regulation. As evidence specific to adolescents remains limited and is not available for all relevant metabolic, microbial and endocrine pathways, studies conducted in younger children, adults and experimental models were included if they provided insights into processes that are not well characterised in adolescents.

The search process yielded approximately 360 records that met the broad thematic scope of the review. After removing duplicates, the titles and abstracts were screened for relevance to adolescent populations or mechanistic pathways pertinent to this age group. This resulted in a refined set of studies for full-text assessment. As evidence specific to adolescents is limited and unevenly distributed across metabolic, microbial and endocrine domains, mechanistic studies involving younger children, adults and experimental models were also considered, provided they offered biologically relevant insights into processes that are not well characterised in adolescents. As this is a narrative review with a structured search strategy rather than a systematic review, no formal risk-of-bias tool was applied; however, methodological clarity and population relevance were considered when interpreting the evidence.

## 3. Human Gut Microbiome: Composition, Development and Functional Roles

### 3.1. The Human Gut Microbiome as a Metabolic and Endocrine Interface

This section synthesises current evidence on the diet–microbiome–hormone interactions that shape early cardiometabolic vulnerability in adolescents, providing the biological foundation for the thematic areas discussed in the subsequent parts of the review. The human gut microbiome comprises a complex community of microorganisms, including bacteria (approximately 90%), archaea (less than 1%), fungi (less than 1%), protozoa and viruses. These microorganisms colonise the gastrointestinal tract [34,35]. The intestinal ecosystem is dominated by the bacterial phyla, *Bacteroidetes* and *Firmicutes*, which together account for around 90% of the total bacterial population. *Actinobacteria* constitute the majority of the remaining Gram-positive taxa [36]. In addition to these dominant groups, minor microbial populations substantially contribute to metabolic cross-feeding networks and the synthesis of bioactive metabolites that influence host physiology. While many of these microorganisms typically function as commensals, changes in environmental or host conditions can cause them to behave in a pathogenic or opportunistic manner [37,38]. Consequently, the gut microbiome is increasingly recognised as an active metabolic and endocrine interface linking diet, environmental exposures, and host regulatory systems, rather than merely as a passive microbial community [39].

The functional importance of this system is further underscored by its genetic capacity, as the gut microbiome is estimated to harbour around three million genes, which is approximately 150 times more than the human genome [40]. This extensive genetic repertoire enables metabolic and biochemical functions that the human host cannot perform independently [41], establishing the microbiome as a ‘second genome’ and a promising source of diagnostic biomarkers and therapeutic targets [42]. Microbial metabolites produced by gut microorganisms act as key molecular signals that shape metabolic balance, immune function and neuroendocrine regulation [43]. The composition and functional capacity of the gut microbiome are shaped to a remarkable extent by dietary exposures, which remain one of the most influential environmental determinants of microbial activity [44]. Through these mechanisms, microbial metabolites regulate insulin sensitivity, lipid metabolism, inflammatory responses and appetite control, thereby linking gut microbial activity to cardiometabolic risk pathways [45].

The composition of the gut microbiota, which begins to form at birth and remains highly dynamic throughout life, is strongly shaped by the mode of delivery, with vaginally delivered infants being primarily colonised by *Lactobacillus* and *Prevotella*, whereas those born via caesarean section acquire predominantly skin-associated bacteria such as *Propionibacterium*, *Staphylococcus* and *Corynebacterium* [46]. Early-life feeding practices also influence microbial development, with distinct microbial profiles observed in breastfed versus formula-fed infants [47]. Other factors that influence the maturation of the microbiome include antibiotic exposure, environmental microbial diversity, geographic location and dietary patterns during childhood [48]. Despite extensive research efforts, a universal definition of a “healthy microbiome” remains elusive as individual variability reflects the complex interactions between genetics, the environment, lifestyle and health status [49]. Consequently, current research is increasingly focusing on functional resilience, microbial diversity, and metabolic stability as more relevant indicators of microbiome health.

A pivotal window of biological maturation during which marked restructuring of the gut microbiome is undergone and an adult-like configuration is progressively attained is represented by adolescence [50]. The hormonal environment of puberty shapes this process by influencing microbial composition, metabolic activity, and immune responsiveness. Increases in sex hormones, growth hormone and insulin-like growth factor 1 (IGF-1) that occur at the same time establish a dynamic bidirectional interaction between the endocrine pathways and gut microbial communities [51]. The presence of different microbial signatures in males and females is a characteristic of this developmental stage. For example, adolescent girls usually have higher levels of Bifidobacterium, while boys have higher levels of *Bacteroides*. These differences appear to be linked to differences in oestrogen and testosterone levels [52]. These hormone-dependent microbial shifts may influence nutrient metabolism, immune maturation and susceptibility to metabolic dysregulation, while also interacting with dietary factors to determine long-term metabolic health [50,51,52].

Maintaining metabolic equilibrium requires both microbiome diversity and functional stability, and a diet low in fibre and high in ultra-processed foods can reduce microbial richness while promoting the production of endotoxins such as lipopolysaccharides (LPS), which impair intestinal barrier integrity and contribute to systemic inflammation—processes strongly implicated in metabolic and cardiovascular diseases [53]. Reduced microbial diversity has also been linked to impaired SCFA production, dysregulated bile acid metabolism, and altered gut–brain signalling pathways that affect appetite regulation and energy balance [7]. Thus, these findings emphasise the pivotal role of the gut microbiome in mediating interactions between environmental factors and host metabolic pathways. Consequently, strategies aimed at modulating microbial composition through targeted dietary interventions, including fermentation-derived functional ingredients, are being increasingly explored as potential tools for preventing early cardiometabolic risk.

### 3.2. Dietary Modulation of the Gut Microbiome and Its Metabolic Consequences in Adolescents

Diet is one of the most powerful environmental factors that shape the composition and functional capacity of the gut microbiome. The distribution of macronutrients (such as dietary fibre, fats and proteins), the proportion of plant-based foods in the diet, and overall dietary patterns (including high-fat and ultra-processed diets) exert a profound influence on microbial ecology and metabolic outputs [54,55]. In addition to diet, factors such as mode of delivery, infant feeding practices, antibiotic exposure, environmental conditions, sleep and stress during the early stages of life contribute to inter-individual variability in microbial composition [56]. Genetic and immunological predispositions also modulate microbial structure and function, highlighting the multifactorial nature of host–microbiome interactions [57]. Importantly, adolescence is a sensitive developmental period during which dietary behaviours can rapidly reshape microbial communities as both the gut microbiota and the host’s metabolic systems are still maturing.

Systemic metabolic regulation, inflammatory signalling and endocrine responses are influenced by metabolites generated during microbial fermentation of indigestible dietary substrates, a process in which fibres and plant polysaccharides serve as the primary biochemical drivers [41,58,59]. Beyond their local effects in the intestine, SCFAs function as signalling molecules that regulate host gene expression, inflammatory responses and endocrine activity [59]. By activating receptors such as GPR41 and GPR43, they enhance insulin sensitivity, reduce triglyceride levels and stimulate the secretion of GLP-1 and PYY. This contributes to the regulation of appetite and improved glucose homeostasis [60]. Thus, microbial fermentation products establish a direct link between dietary composition and the hormonal regulation of energy balance and metabolic homeostasis.

By contrast, dietary patterns characterised by a high intake of saturated fats, refined sugars and ultra-processed foods have been linked to reduced microbial diversity and an increased prevalence of pro-inflammatory microbial species, and such diets may also promote intestinal barrier dysfunction, endotoxin (lipopolysaccharide, LPS) translocation and chronic low-grade inflammation, which are processes strongly associated with metabolic disorders and early cardiovascular risk [61]. These alterations are also associated with impaired SCFA production, disrupted bile acid metabolism, and dysregulation of host lipid and glucose homeostasis [62]. In adolescents, these mechanisms are of particular concern, as metabolic programming during this developmental period can have long-lasting effects on cardiometabolic health in adulthood [1].

Therefore, dietary modulation of the gut microbiome is a key way in which nutritional factors influence metabolic health. Patterns of metabolic resilience or vulnerability can be traced to dietary habits, with fibre-rich, plant-based and fermented foods supporting diverse microbial ecosystems, whereas highly processed diets are consistently linked to communities associated with inflammation and metabolic imbalance [30,53,63]. Consequently, it is essential to understand how specific dietary components shape microbial metabolism in order to develop targeted nutritional strategies aimed at preventing early cardiometabolic disturbances during adolescence.

### 3.3. Precision Fermentation and Adolescent Cardiovascular Risk

The introduction of PF at this stage of the review highlights its reliance on the metabolic, microbial and endocrine pathways described in the preceding sections. These pathways collectively define the biological context in which this technology can impact early cardiovascular risk in adolescents. PF is a biotechnological process that uses genetically engineered microorganisms to produce specific bioactive compounds, and, unlike conventional fermentation-which relies on naturally occurring microbial metabolic activity—PF employs characterised microbial strains and controlled bioprocessing systems to synthesise defined molecules with targeted biological functions [27]. This technology has expanded the range of food-derived ingredients capable of modulating the diet–microbiome–hormone axis, which is directly relevant to the early identification and modulation of cardiovascular risk factors in adolescents, and, by enabling the scalable and reproducible production of bioactive molecules, PF creates new opportunities for developing functional foods that influence metabolic and endocrine pathways associated with cardiometabolic health [64]. In this section, PF is presented as a natural extension of the diet–microbiome–hormone framework set out earlier. It provides a technology-based approach that is based directly on the metabolic and endocrine pathways described in previous parts of the manuscript.

It was known that one of the most advanced applications of PF is the production of bioactive peptides with cardioprotective properties, including recombinant analogues of casein- and whey-derived peptides such as isoleucine-proline-proline (IPP) and valine-proline-proline (VPP), which act as inhibitors of angiotensin-converting enzyme (ACE) [65,66,67]. By reducing the formation of angiotensin II, these peptides decrease vascular smooth muscle contraction and enhance the production of endothelial nitric oxide (NO). Consequently, ACE inhibition promotes vasodilation, improves endothelial responsiveness and reduces peripheral vascular resistance. These mechanisms contribute to measurable reductions in both systolic and diastolic blood pressure by modulating the renin–angiotensin–aldosterone system (RAAS) [68,69]. PF enables the production of these peptides at a high level of purity, free from lactose and dairy allergens. This makes them suitable for incorporation into functional foods and beverages aimed at preventing early hypertension in adolescents [70]—a condition that is becoming increasingly prevalent in populations adopting Western dietary patterns that are high in saturated fats and sodium. Therefore, PF-derived peptides are promising candidates for nutraceutical products targeting the prevention of early vascular dysfunction [66].

A second major application involves prebiotic oligosaccharides and postbiotic metabolites that are engineered through PF and which directly modulate the gut microbiome and its endocrine outputs. Precision-designed human milk oligosaccharides (HMOs), galacto-oligosaccharides (GOS) and SCFA-enhancing substrates can selectively stimulate the growth of beneficial microbial species, such as *Bifidobacterium* and *Akkermansia* [71,72]. SCFAs, particularly butyrate and propionate, play a central role in metabolic regulation. They do this by enhancing intestinal barrier integrity, reducing systemic inflammation, improving insulin sensitivity and modulating appetite-regulating hormones such as GLP-1 and PYY. These metabolites act through G-protein-coupled receptors (e.g., GPR41 and GPR43) and epigenetic mechanisms, thereby influencing host metabolic signalling pathways [73,74]. These effects counteract the dysbiosis, reduced SCFA production and hormonal disturbances induced by Western diets, which are typically high in refined sugars and low in fermentable fibres. PF-derived prebiotics therefore represent a targeted strategy for improving lipid metabolism, reducing hepatic VLDL secretion and lowering early atherogenic risk in adolescents. By restoring microbial diversity and metabolic cross-feeding within the gut ecosystem, these compounds help re-establish a balanced microbiome–host metabolic interface [75].

**Figure 1:**
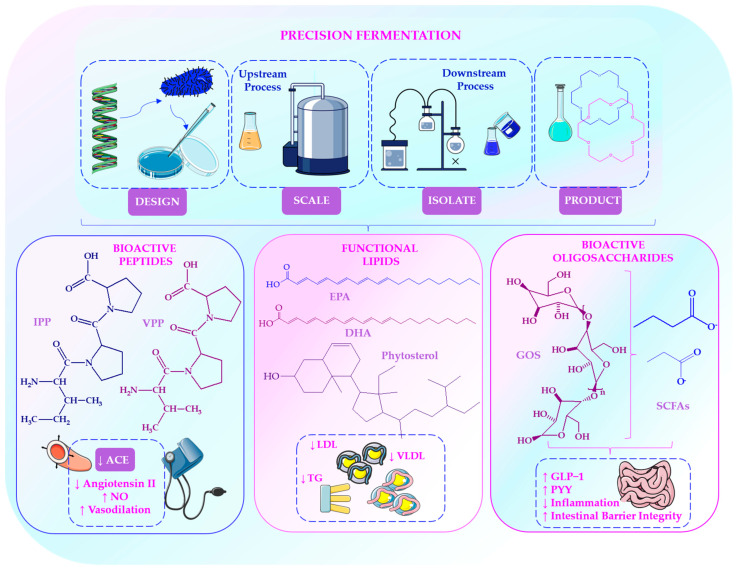
Mechanisms of action of food ingredients produced by precision fermentation (PF) methods on metabolic and vascular health. Genetically modified microorganisms enable the production of bioactive peptides (e.g., IPP, VPP) that inhibit ACE, reduce angiotensin II production, improve endothelial function, and lower blood pressure. Precisely designed oligosaccharides (HMO, GOS) and postbiotic metabolites increase the abundance of beneficial bacteria (*Bifidobacterium*, *Akkermansia*), support SCFA production, and modulate gut hormones (GLP-1, PYY) via GPR41/43 receptors. Lipids and sterols produced in PF, including EPA, DHA, and phytosterols, improve lipid profiles, reduce triglyceride synthesis, and lower LDL levels. Together, PF components strengthen the intestinal barrier, reduce LPS translocation, lower inflammation, and counteract early vascular dysfunction induced by a Western diet in adolescents. Image provided by Servier Medical Art (https://smart.servier.com, 6 April 2026), licensed under CC BY 4.0 (https://creativecommons.org/licenses/by/4.0/, accessed on 9 April 2026). Abbreviations: ACE—angiotensin-converting enzyme; DHA—docosahexaenoic acid; EPA—eicosapentaenoic acid; GLP-1—glucagon-like peptide-1; GOS—galacto-oligosaccharides; IPP—isoleucine-proline-proline; LDL—low-density lipoprotein; NO—nitric oxide; PYY—peptide YY; SCFAs—short-chain fatty acids; TG—triglycerides; VLDL—very-low-density lipoprotein; VPP—valine-proline-proline.

Figure 1 shows how food ingredients produced through PF can affect metabolic and vascular processes. The figure shows the proposed effects on blood pressure, lipid metabolism, the composition of the gut microbiota and inflammatory pathways. These pathways are presented as potential contributors to endothelial function and cardiometabolic health. It should be noted, however, that direct clinical evidence in adolescents remains limited. The figure summarises findings from various sources. Pathways supported by data from adolescents are described in the main text. Mechanisms identified in adult cohorts, younger paediatric populations, or experimental models (animal or in vitro) are also included to provide biological context for processes that have not yet been fully characterised in adolescents.

In addition, PF is increasingly being used to produce fermentative lipids and sterols that have well-established cardiovascular benefits [76]. The microbial synthesis of omega-3 fatty acids, such as eicosapentaenoic acid (EPA) and docosahexaenoic acid (DHA), provides a sustainable alternative to marine sources while maintaining equivalent biological activity [77]. Engineered yeasts and microalgae enable the efficient biosynthesis of long-chain polyunsaturated fatty acids, thereby reducing reliance on fish-derived oils and the associated environmental pressures [78,79]. EPA and DHA reduce hepatic triglyceride synthesis, stabilise cardiomyocyte membranes and modulate inflammatory eicosanoid pathways [80]. Concurrently, precision-fermented phytosterols inhibit intestinal cholesterol absorption by competing with dietary sterols at the micellar level, thereby reducing low-density lipoprotein (LDL) cholesterol levels [81]. These PF-derived lipid products can be incorporated into plant-based foods to address common nutritional deficiencies in Western adolescent diets, which are often characterised by low omega-3 intake and high pro-inflammatory fat consumption. Consequently, PF-derived lipid ingredients may improve lipid profiles and reduce long-term cardiovascular risk [82].

Finally, PF enables the production of purified postbiotics and microbial metabolites, including signalling molecules derived from butyrate, propionate and lactate [26]. These molecules have direct effects on vascular and metabolic health. These compounds enhance intestinal barrier integrity, reduce endotoxin (LPS) translocation and modulate bile acid signalling pathways that are involved in cholesterol metabolism and glucose homeostasis [83,84]. By regulating inflammatory responses and endothelial signalling pathways, postbiotics improve vascular function and metabolic resilience [85]. This grounded action is particularly relevant for adolescents who consume Western diets, as these dietary patterns increase gut permeability, elevate inflammatory markers and accelerate early vascular dysfunction [86,87]. Therefore, precision fermentation is a promising approach for developing next-generation functional food ingredients that target the microbiome–metabolism–hormone axis involved in early cardiometabolic programming.

Taken together, these applications establish precision fermentation as a technology that broadens the range of applications for fermentation-derived ingredients, providing a logical link to the tiered framework discussed in the next section.

### 3.4. Fermentation-Derived Products as a Three-Tier Framework for Modulating the Diet–Microbiome–Hormone Axis

A growing body of evidence shows that the functional outputs of fermentation can be conceptualised as three complementary and hierarchically organised tiers that determine how fermentation-derived inputs shape host metabolic and hormonal responses, with the first tier encompassing fermented foods as direct dietary sources of exogenous bioactive metabolites, as traditionally fermented products-including yoghurt, kefir, kimchi, sauerkraut, kombucha, tempeh, fermented soy products, fermented milk preparations and fermented grain-based foods-contain viable microbial communities together with a diverse array of pre-formed bioactive compounds generated during microbial metabolism, such as short-chain organic acids, bacteriocins, biogenic amines, vitamins, antioxidants and bioavailable micronutrients [88,89].

These products commonly contain microorganisms belonging to the genera *Lactobacillus*, *Bifidobacterium*, *Lactococcus*, *Leuconostoc* and *Saccharomyces*, which contribute to intestinal homeostasis, immune regulation and barrier integrity through the production of organic acids, antimicrobial compounds and signalling metabolites [89]. When consumed, fermented foods deliver exogenous metabolites directly to the gastrointestinal tract, where they can influence luminal chemistry, modulate the resident microbiota and interact with host epithelial and enteroendocrine cells. In adolescents, whose gut microbiome is still maturing and particularly responsive to dietary inputs, regular consumption of fermented foods has been associated with enhanced microbial diversity, reduced inflammatory markers, improved lipid and glucose metabolism, and better metabolic outcomes, each of which has direct relevance to early cardiovascular risk [1,30]. Experimental and clinical evidence further indicates that fermented foods may support SCFA production, improve intestinal microbial balance and modulate host metabolic signalling pathways involved in cardiometabolic regulation [89].

The second tier concerns microbial fermentation products acting as metabolic mediators, a category that overlaps substantially with the emerging concept of postbiotics. These include SCFAs, secondary bile acids, indole derivatives, equol and other microbially produced signalling molecules that arise from the in situ fermentation of dietary substrates in the gut [26,38]. As detailed in the preceding sections, these metabolites function at the intersection of microbial and host metabolism: they modulate intestinal barrier integrity, regulate GLP-1 and PYY secretion via GPR41/GPR43, influence RAAS activity through ACE-inhibitory peptides and interact with the GH/IGF-1 axis and sex steroid metabolism [10,84,90]. Importantly, several traditional fermented foods have been associated with increased abundance of SCFA-producing bacterial genera, including *Faecalibacterium* and *Roseburia*, alongside reductions in inflammatory markers and obesity-related metabolic disturbances [89]. Fermented milk products enriched with *Lactococcus lactis*, as well as synbiotic formulations containing inulin and *Lactobacillus* strains, have additionally demonstrated beneficial effects on blood pressure, serum lipids and intestinal microbial homeostasis, supporting the concept that fermentation-derived metabolites function as endocrine and metabolic signalling mediators within the gut–host axis [89]. Within the framework of this review, this tier represents the core of the diet–microbiome–hormone axis, as it is through these microbially produced mediators that dietary composition is translated into endocrine and vascular signals relevant to cardiometabolic health in adolescents. Dysbiosis, by reducing the diversity and abundance of fermenting taxa responsible for generating this metabolite landscape, directly impairs these signalling pathways and thereby contributes to the early cardiometabolic risk phenotype described in Section 4.

The third tier introduces a qualitatively distinct level of intervention: the precision-engineered production of bioactive compounds through PF. As described in Section 3.3, PF uses genetically characterised microorganisms operating under controlled bioprocessing conditions to synthesise defined molecules with targeted biological activity [26,27]. Unlike the first two tiers, which depend on the metabolic activity of naturally occurring or resident microbial communities, PF enables the scalable and reproducible biosynthesis of molecules that cannot be obtained in sufficient quantity or purity through conventional fermentation. These include recombinant ACE-inhibitory peptides, precision-designed HMOs and GOS, microbially synthesised omega-3 fatty acids, phytosterols and purified postbiotic preparations with defined structural and functional properties [66,71,78]. By enabling the design of functional food ingredients whose biological activity is matched to specific pathways in the diet–microbiome–hormone axis, PF represents a precision nutritional strategy that complements and extends the first two tiers.

These three tiers form an integrated framework that positions fermentation-derived products not merely as food ingredients but as a coherent set of interventional tools acting across different scales of biological complexity, from the food matrix and gut lumen, through microbial metabolic networks, to precisely engineered molecular outputs. Thus, evidence from traditional fermented foods and modern precision fermentation approaches supports the concept that fermentation-derived products can modulate microbial ecology, endocrine signalling, immune responses and vascular function through interconnected mechanisms operating along the diet–microbiome–hormone axis. This hierarchical structure underpins the rationale for evaluating fermentation as a strategy for reducing early cardiometabolic risk in adolescents and provides the conceptual scaffold for the evidence reviewed in subsequent sections.

### 3.5. Fermentable Substrates and Microbial Metabolism

Adequate dietary fibre intake promotes the growth of beneficial bacteria, such as *Bifidobacterium* and *Faecalibacterium*, which are important contributors to fermentative metabolism and SCFA production [91,92]. Higher abundances of *Faecalibacterium*, *Prevotella* and *Bacteroides* have been associated with better glycaemic control, reduced adiposity, improved bowel function and lower systemic inflammation [93]. *Faecalibacterium prausnitzii*, in particular, is recognised as one of the main butyrate-producing bacteria and plays an important role in maintaining intestinal barrier integrity and anti-inflammatory homeostasis [94]. These observations emphasise the importance of fermentable substrates in shaping microbial metabolism and influencing host metabolic outcomes. In adolescents, whose metabolic and hormonal systems are still developing, sufficient intake of such substrates may be especially important for maintaining microbiome stability and supporting metabolic homeostasis.

Evidence indicates that Western-style diets characterised by high intakes of saturated fats, refined sugars and ultra-processed foods are associated with reduced microbial diversity, increased abundance of proteolytic and pro-inflammatory taxa and a decline in fibre-fermenting bacteria, creating a dysbiotic state that diminishes the capacity for beneficial microbial fermentation and promoting intestinal barrier dysfunction, thereby facilitating the translocation of endotoxins such as LPS into the bloodstream and triggering chronic low-grade inflammation and metabolic endotoxemia, alterations that are of particular concern during adolescence because prolonged exposure to inflammatory and metabolically unfavourable conditions may contribute to early cardiometabolic programming and increase lifetime cardiovascular risk [95,96,97].

Furthermore, diets high in fat and sugar may promote the proliferation of Gram-negative bacteria and increase exposure to microbial-associated molecular patterns. For example, peptidoglycan can activate the NOD1 signalling pathway, contributing to inflammation, insulin resistance and hyperglycaemia in obesity [98]. NOD1 expression is also modulated by the gut microbiota, further amplifying host inflammatory responses [99]. At the same time, the depletion of beneficial fermenting taxa fosters a pro-inflammatory metabolic environment associated with hypertension, dyslipidaemia and insulin resistance—key risk factors for cardiovascular disease [100]. Collectively, these findings indicate that fermentable substrates shape microbial ecology and determine the profile of metabolites produced within the intestinal environment.

### 3.6. SCFAs as Metabolic, Immunological and Endocrine Mediators

Among the metabolites produced during microbial fermentation, SCFAs have emerged as central mediators linking gut microbial activity with host metabolic and endocrine regulation [101,102]. Experimental evidence indicates that butyrate supports colonocyte energy metabolism and intestinal barrier stability, whereas acetate and propionate contribute to systemic control of lipid and glucose balance [103,104]. Many of these effects are now understood to operate through G protein-coupled receptors such as GPR41 and GPR43, whose activation enhances insulin sensitivity, lowers circulating triglycerides and stimulates enteroendocrine secretion of GLP-1 and PYY [90]. These hormones play a key role in appetite regulation, satiety and glucose handling, providing a mechanistic connection between microbial fermentation and endocrine pathways governing energy balance [90]. This signalling axis appears particularly relevant during adolescence, a developmental period characterised by rapid shifts in energy demand, hormonal activity and appetite control.

Systemic inflammatory tone, hepatic lipid handling and adipose tissue signalling are influenced by shifts in host gene expression that arise partly from SCFA-dependent inhibition of histone deacetylases [105]. These metabolites also temper immune activation by reshaping the activity of key immune cell populations and attenuating pro-inflammatory pathways. By strengthening epithelial tight junction architecture and reducing paracellular permeability, SCFAs limit the translocation of endotoxin-rich luminal components, thereby reducing the risk of metabolic endotoxemia [106]. Through these combined actions, they contribute to the preservation of intestinal barrier integrity and the maintenance of balanced immune responses.

These effects counteract metabolic disturbances associated with Western dietary patterns, including dysbiosis, impaired barrier function, insulin resistance, endothelial dysfunction and chronic low-grade inflammation. Therefore, strategies aimed at enhancing SCFA production or activity are increasingly recognised as promising approaches for modulating the diet–microbiome–hormone axis [107]. This is particularly important in adolescents, whose gut microbiome, endocrine system and metabolic pathways remain highly responsive to dietary and environmental inputs.

A growing body of evidence shows that, from the perspective of precision fermentation, SCFA-related pathways represent well-defined biochemical targets for next-generation functional ingredients, as precision-fermented bioactive compounds may enhance beneficial microbial fermentation, deliver postbiotic metabolites, support intestinal barrier integrity or selectively stimulate signalling pathways involved in metabolic and hormonal regulation, thereby establishing that understanding SCFA-mediated mechanisms provides a strong foundation for identifying fermentation-derived and precision-fermented compounds capable of reducing early cardiometabolic risk in adolescents.

## 4. Physiological, Hormonal and Microbiome-Related Determinants of Early Cardiovascular Risk in Adolescents

### 4.1. The Microbiome–Appetite–Energy Regulation Axis

The gut microbiota plays a central role in regulating appetite and energy balance, partly by influencing ghrelin, the primary orexigenic hormone responsible for stimulating hunger [108]. Appetite regulation is governed by the complex interactions between the gastrointestinal tract, the central nervous system and endocrine signalling pathways. These interactions are collectively referred to as the gut–brain axis [109]. There is increasing evidence that the microbiota can modulate ghrelin secretion and signalling directly and indirectly. For example, this can be achieved via microbial metabolites such as SCFAs, or through interactions with neural pathways within the gut–brain axis [110,111]. In particular, gut microorganisms influence enteroendocrine cells, which act as intermediaries between the digestive system and neuroendocrine signalling networks. Alterations in microbial composition may also affect ghrelin signalling at the growth hormone secretagogue receptor (GHS-R) level, as well as circulating ghrelin concentrations [110,112]. These mechanisms demonstrate how the microbiome integrates metabolic and neuronal signals to control hunger and appetite, particularly during adolescence, a period characterised by rapid growth and elevated energy requirements. Disruptions in these pathways may therefore have significant consequences for eating behaviour and long-term metabolic health.

This relationship has been supported by both clinical and population-based studies. Dysbiosis, which is defined as an imbalance in the composition of microbes, has been linked to increased ghrelin levels and impaired appetite control. Individuals with dysbiosis often experience increased hunger and higher energy intake, which can lead to obesity and related metabolic disorders [113]. Furthermore, these alterations can affect other hormonal regulators of energy balance, including leptin signalling, thereby disrupting appetite regulation and metabolic homeostasis [114]. Experimental studies in animal and human models provide additional insight, demonstrating that shifts in microbial composition influence the production of SCFAs and other metabolites that regulate ghrelin secretion and its downstream metabolic effects [115,116]. These findings suggest that microbial metabolic activity acts as a key intermediary linking dietary patterns with endocrine signals that control hunger and energy intake.

The way in which SCFAs contribute to metabolic regulation is partly through the modulation of insulin sensitivity via receptors that are involved in energy balance. These receptors include GPR41 and GPR43 [90]. They also limit low-grade inflammation and the translocation of endotoxin-rich luminal components by reinforcing epithelial tight junctions and reducing intestinal permeability, thereby supporting intestinal homeostasis [104,117]. These metabolites act as molecular signals that connect microbial activity with central and peripheral pathways that govern appetite, satiety, and energy utilisation. When produced in adequate amounts, SCFAs stimulate enteroendocrine cells to release GLP-1 and PYY, which are hormones that slow gastric emptying, enhance satiety, and improve postprandial glucose handling [117]. These coordinated effects help SCFAs to maintain the integrity of metabolic signalling networks, which are often disrupted in adolescents who consume a Western-style diet [118].

**Figure 2:**
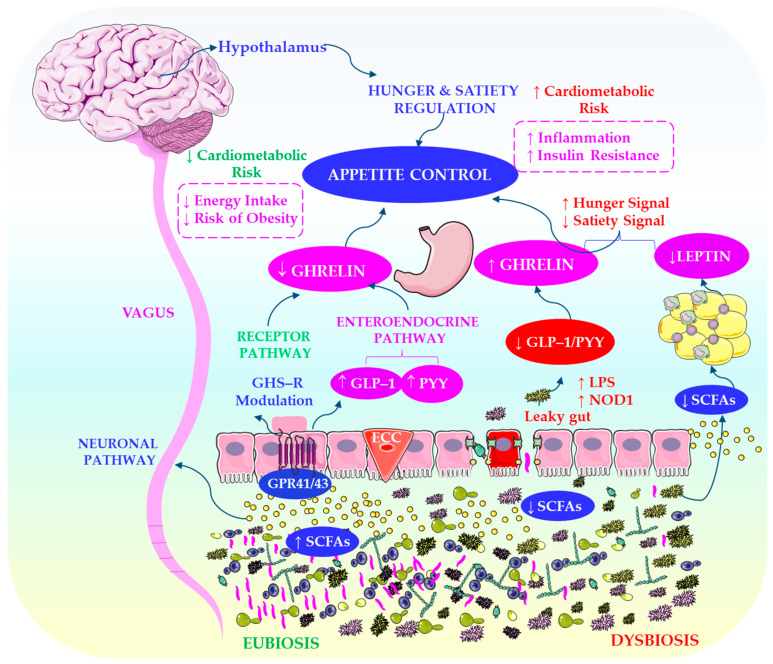
The influence of gut microbiota and microbial metabolites on the regulation of ghrelin, appetite and energy balance. Changes in the microbiota composition influence ghrelin secretion and its signaling via the GHS-R receptor, modulating hunger and eating behavior during adolescence. Short-chain fatty acids (SCFAs), produced by gut bacteria, stimulate enteroendocrine cells to release satiety hormones (GLP-1, PYY), strengthen the integrity of the intestinal barrier, and activate GPR41/43 receptors, integrating metabolic signals from the gut–brain axis. Dysbiosis increases ghrelin levels, disrupts appetite control, and promotes excessive energy intake, which may lead to obesity and early metabolic disorders. Microbial metabolites also influence the vagus nerve and hypothalamic centers, linking microbiome activity to the central regulation of hunger and satiety. Image provided by Servier Medical Art (https://smart.servier.com, 6 April 2026), licensed under CC BY 4.0 (https://creativecommons.org/licenses/by/4.0/, accessed on 9 April 2026). Abbreviations: ECC—enteroendocrine cells; GHS-R—growth hormone secretagogue receptor; GPR41/43—G-protein–coupled receptor 41/43; SCFAs—short-chain fatty acids; GLP-1—glucagon-like peptide-1; PYY—peptide YY; LPS—lipopolysaccharide; NOD1—nucleotide-binding oligomerization domain-containing protein 1.

Figure 2 illustrates how the gut microbiota and their metabolites regulate ghrelin secretion, appetite and energy balance via the gut–brain axis. It emphasises the significance of SCFAs, enteroendocrine signalling and neural pathways in preserving metabolic homeostasis, and the consequences of dysbiosis for appetite control and obesity risk.

In addition to metabolic signalling, gut bacteria modulate communication along the gut–brain axis, thereby shaping feeding behaviour and hunger perception [119]. Microbial metabolites can influence vagal nerve activity and central appetite-regulating centres in the hypothalamus, effectively linking intestinal microbial activity with the neural control of food intake [120]. Clinical observations, such as changes in ghrelin levels following *Helicobacter pylori* eradication therapy, further support the concept that alterations to the microbiome can directly affect appetite-related hormones [121]. Three principal mechanisms have been proposed to explain microbiome-mediated regulation of ghrelin: (i) microbial metabolites acting on enteroendocrine cells; (ii) neural signalling via the gut–brain axis; and (iii) modulation of hormone receptor expression, including ghrelin receptors [110]. These pathways illustrate the multifactorial role of the gut microbiota in regulating appetite and energy homeostasis during adolescence. A deeper understanding of these interactions is essential for developing targeted nutritional strategies, particularly those involving precision-fermented functional ingredients, that aim to restore balanced appetite signalling and reduce early cardiometabolic risk.

### 4.2. Microbiome-Driven Inflammation, Insulin Resistance and Early Metabolic Dysfunction

Clinical observations indicate that adolescent obesity is strongly associated with an increased risk of insulin resistance, a metabolic disturbance that markedly elevates the likelihood of developing cardiovascular disease in adulthood, and it is increasingly recognised that during this critical developmental period, rapid physiological growth and hormonal changes heighten metabolic demands and may amplify the impact of metabolic imbalances, making adolescents particularly susceptible to cardiometabolic dysregulation [122,123]. A substantial body of evidence indicates that adolescent obesity is closely linked to gut microbiota dysbiosis, which promotes chronic low-grade inflammation, excessive weight gain, lipid abnormalities and impaired glucose tolerance [87,124]. This dysbiotic state is characterised by reduced microbial diversity and decreased abundance of fibre-fermenting bacteria that are beneficial, as well as an increased prevalence of pro-inflammatory taxa [125]. Notably, interventions that restore microbial balance, particularly through dietary modifications or targeted, microbiome-based strategies, have been demonstrated to alleviate these metabolic disturbances and reduce long-term cardiometabolic risk [126].

Recent research highlights that the gut microbiota plays a pivotal role in regulating inflammatory processes by producing SCFAs such as butyrate and propionate, which influence immune function as well as glucose and lipid metabolism, and experimental studies have demonstrated that these metabolites modulate inflammatory signalling pathways by inhibiting histone deacetylases (HDACs) and activating G protein-coupled receptors including GPR41 and GPR43, thereby regulating immune cell activity and maintaining metabolic homeostasis [127]. However, disruption to the balance of gut microbes can lead to systemic inflammation, metabolic dysfunction and insulin resistance—key early indicators of cardiovascular disease [128]. One central mechanism underlying this process is metabolic endotoxemia, whereby LPS derived from Gram-negative bacteria enter the bloodstream due to increased intestinal permeability. This triggers inflammatory cascades that impair insulin signalling pathways [129].

It is increasingly apparent that reducing long-term cardiometabolic risk in younger individuals is achievable through strategies that target early disturbances in gut–host metabolic communication [130]. Evidence from studies of adolescents shows that faecal microbiota transplantation, structured lifestyle programmes, and selected probiotic or prebiotic formulations can improve metabolic and hormonal parameters. This indicates that the adolescent microbiome retains substantial plasticity and responsiveness to intervention [131,132]. Restoring balanced interactions between intestinal microbes, endocrine pathways, and metabolically active tissues (including the liver and adipose tissue) depends partly on the efficient fermentation of dietary substrates by microbes, which strengthens epithelial barrier function and supports coordinated metabolic signalling [126]. These benefits are further reinforced by shifting dietary patterns towards higher fibre intake and reduced consumption of ultra-processed foods. Such changes improve insulin sensitivity and attenuate inflammatory activity. These adaptations emphasise the pivotal role of nutrition-driven modulation of the gut ecosystem in stabilising metabolic pathways during adolescence.

Thus, determining which dietary components and functional ingredients can restore microbial balance and reduce inflammation has become a key research priority in preventing adolescent cardiometabolic disorders. In this context, bioactive compounds derived from precision fermentation—including postbiotics and targeted prebiotic substrates—offer a promising way to modulate microbiome-driven metabolic pathways in a controlled, sustainable, and clinically relevant manner [133].

### 4.3. Microbiome-Derived Metabolites and Endothelial Dysfunction

Microbiome-derived metabolites, such as trimethylamine N-oxide (TMAO) and SCFAs, play a significant role in modulating inflammation and endothelial function. This influences the development of atherosclerosis and other cardiovascular disturbances [134]. While TMAO is generally considered pro-atherogenic [135], SCFAs predominantly exert protective effects, such as enhancing endothelial function, reducing oxidative stress and modulating inflammatory signalling pathways [136]. Reduced microbial diversity, a hallmark of dysbiosis, has been associated with hypertension, insulin resistance and other cardiovascular risk factors [137]. In adolescents, early alterations in microbiome composition and metabolite profiles may predispose individuals to subclinical endothelial dysfunction, highlighting the importance of early dietary and microbiome-targeted interventions. Consequently, microbiome-related metabolites, particularly TMAO, are increasingly recognised as promising biomarkers of cardiometabolic risk in this population [138].

A growing body of evidence indicates that TMAO promotes key processes involved in the pathogenesis of atherosclerosis, including macrophage activation, oxidative stress and endothelial dysfunction [139]. TMAO impairs the activity of endothelial nitric oxide synthase (eNOS), thereby reducing the bioavailability of nitric oxide (NO) [140]. TMAO also activates pro-inflammatory signalling pathways such as protein kinase C (PKC) and nuclear factor kappa B (NF-κB) and increases the production of inflammatory cytokines including IL-1, IL-18 and TNF-α [141]. Furthermore, TMAO enhances vascular cell adhesion molecule-1 (VCAM-1) expression to promote monocyte adhesion and inhibits reverse cholesterol transport, while also increasing proprotein convertase subtilisin/kexin type 9 (PCSK9) levels to facilitate low-density lipoprotein (LDL) accumulation [135,142]. In macrophages, TMAO upregulates CD36 receptor expression to promote foam cell formation and increases ADP-induced platelet reactivity [139]. These mechanisms exacerbate vascular inflammation, impair endothelial function, and accelerate atherosclerotic plaque development. This establishes a link between diet, microbiome dysbiosis, and early cardiovascular risk in adolescents [135].

By contrast, SCFAs—particularly butyrate and propionate—counteract many of these harmful effects. They enhance eNOS activity and increase NO bioavailability, reduce oxidative stress, inhibit NF-κB activation and suppress the release of pro-inflammatory cytokines [118]. Furthermore, by strengthening the integrity of the endothelial and intestinal barriers, SCFAs reduce vascular permeability and limit the translocation of endotoxins, such as lipopolysaccharide (LPS), into the circulation [143]. These findings highlight the dual roles of microbiome-derived metabolites: balancing harmful compounds such as TMAO with protective metabolites such as SCFAs may be critical for endothelial health and early cardiovascular risk in adolescents [119].

Table 1 summarises key in vitro and in vivo studies illustrating the pathways linking dietary patterns, microbial metabolites, hormonal regulation and cardiometabolic outcomes in adolescents and related experimental models. Table 1 includes studies representing a broad spectrum of evidence sources, ranging from epidemiological and clinical data in adolescents to observational research in younger paediatric or adult populations, as well as experimental findings derived from animal models, transgenic strains and in vitro systems. These categories reflect the current state of the field, where research focusing on adolescents remains limited and is distributed unevenly across metabolic, microbial, and endocrine domains. The table therefore distinguishes between human observational or interventional studies, experimental in vivo models and in vitro approaches, allowing readers to interpret each finding in relation to its methodological context and evidentiary strength.

### 4.4. Microbiome-Sex Hormone Interactions During Adolescence

The role of the gut microbiome in modulating sex steroid hormones is becoming increasingly recognised. Sex steroids, including oestrogens and androgens, engage in bidirectional interactions with the gut microbiota. On the one hand, microbial composition can influence circulating hormone levels through enzymatic activity. On the other hand, sex hormones can shape microbial ecology by promoting or suppressing specific taxa [155]. For example, microbial enzymes such as β-glucuronidases, sulfatases, and hydroxysteroid dehydrogenases facilitate the deconjugation and reactivation of steroid hormones in the enterohepatic circulation, thereby affecting their bioavailability and metabolic activity [156,157]. These interactions are particularly relevant during adolescence, a period characterised by a rapid increase in sex hormone levels and the development of sex-specific metabolic patterns.

Oestrogens appear to promote a more favourable microbial profile characterised by an increased abundance of bacteria that produce SCFAs with anti-inflammatory, anti-atherogenic and metabolically protective properties [158]. In oestrogen-dominant individuals, enhanced SCFA production may support endothelial function, improve insulin sensitivity, and optimise lipid metabolism, which could explain the lower cardiovascular risk observed in premenopausal females [159,160]. In contrast, androgens may influence lipid metabolism through microbiome-mediated pathways by modulating the production of microbial metabolites, including TMAO, bile acids, and branched-chain amino acids. These metabolites affect host lipid handling, insulin sensitivity, and systemic inflammatory responses [161,162]. Consequently, microbiome–hormone interactions contribute to sex-specific trajectories of cardiometabolic risk that begin to emerge during adolescence.

**Figure 3:**
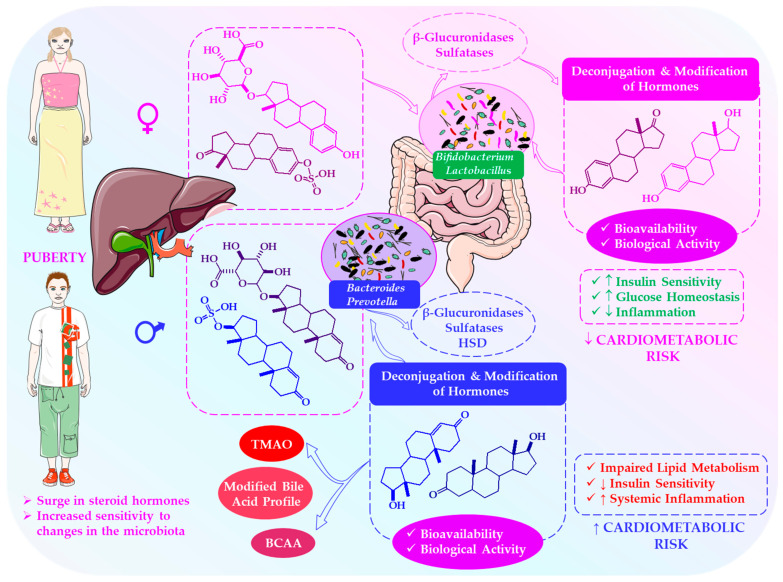
Bidirectional interactions between sex hormones and the gut microbiome influence metabolic regulation and early cardiometabolic risk. Microbial enzymes (β-glucuronidases, sulfatases, hydroxysteroid dehydrogenases) modulate enterohepatic circulation of steroid hormones, altering the bioavailability of estrogens and androgens. Estrogens promote a microbiome enriched in SCFA, producing bacteria (*Bifidobacterium*, *Lactobacillus*), supporting anti-inflammatory activity, endothelial function, insulin sensitivity, and lipid metabolism. Androgens shape microbial composition toward taxa influencing lipid metabolism and inflammatory pathways (*Bacteroides*, *Prevotella*), partly via metabolites such as TMAO, bile acids, and branched-chain amino acids. These sex-specific microbiome–hormone interactions contribute to the divergent cardiometabolic trajectories that emerge during adolescence. Image provided by Servier Medical Art (https://smart.servier.com, 6 April 2026), licensed under CC BY 4.0 (https://creativecommons.org/licenses/by/4.0/, accessed on 9 April 2026). Abbreviations: BCAA—branched-chain amino acids; HSD—hydroxysteroid dehydrogenase; TMAO—trimethylamine N-oxide.

Figure 3 illustrates the bidirectional interactions between sex hormones and the gut microbiome, as well as their combined effect on metabolic regulation. It emphasises how this crosstalk shapes inflammation, lipid metabolism and insulin sensitivity, thereby contributing to sex-specific cardiometabolic risk during adolescence.

Evidence from animal studies and clinical observations indicates that oestrogen and testosterone influence the composition of gut microbes, with sex-specific differences in dominant bacterial species reflecting different hormonal environments [163,164]. For instance, adolescent females typically have higher levels of *Bifidobacterium* and *Lactobacillus*, which are linked to SCFA production and anti-inflammatory properties [165]. In contrast, males more frequently display increased levels of *Bacteroides* and *Prevotella*, which are linked to lipid metabolism and modulation of inflammatory responses [166]. These differences in microbiome composition may contribute to the early divergence of cardiovascular risk profiles between the sexes [167]. These findings emphasise the significance of microbiome–sex hormone interactions in shaping metabolic health during adolescence. They also emphasise the potential of targeted nutritional strategies, such as the use of precision-fermented bioactive compounds, to modulate microbiome–hormone interactions and reduce early cardiometabolic risk in a sex-specific manner.

### 4.5. Microbiome Interactions with the GH/IGF-1 Axis

The gut microbiome regulates a wide range of metabolic pathways in the host, including those involved in energy homeostasis, glucose and lipid metabolism, and bile acid turnover [168]. Its role extends beyond metabolic processes to encompass physical and psychological health, reflecting its function as a central integrator of neuroendocrine and immunometabolic signalling [169]. Comparative studies consistently demonstrate that individuals with obesity or metabolic syndrome have different microbial compositions to lean individuals, highlighting the strong link between dysbiosis and metabolic dysfunction [93,126]. These differences are often accompanied by altered production of key microbial metabolites, including SCFAs, branched-chain amino acids and secondary bile acids. These metabolites may directly or indirectly influence the growth hormone/insulin-like growth factor-1 (GH/IGF-1) axis [91]. Diet is one of the most important factors in modulating the structure and function of the gut microbiome, thereby shaping metabolite profiles that affect host physiology [6].

In this context, the gut microbiome is increasingly being considered a potential population-level biomarker of health status and exposure to lifestyle-related risk factors. Microbiome profiling, which is usually based on analysing stool samples, is a minimally invasive and cost-effective way of monitoring health in large populations, identifying individuals at increased metabolic risk and evaluating the effect of dietary, lifestyle or therapeutic interventions [170,171]. This approach is particularly relevant during adolescence, as microbial signatures can reflect early-life exposures, dietary habits and ongoing hormonal maturation. This provides insight into future cardiometabolic trajectories. As these signatures integrate metabolic predispositions with cumulative lifestyle influences, they could facilitate the early identification of conditions such as obesity, dyslipidaemia, impaired growth and other metabolic disturbances [1,172].

These broader observations are closely linked to specific endocrine pathways, particularly the GH/IGF-1 axis, which plays a fundamental role in growth, energy balance, and metabolic regulation. Emerging evidence suggests that the gut microbiota interacts with this axis through multiple mechanisms [173]. Microbial metabolites, especially SCFAs such as butyrate and propionate, can enhance IGF-1 synthesis and signalling, thereby supporting lean body mass development, efficient energy metabolism, and vascular health [174]. Additionally, the microbiota influences the secretion of key hormonal regulators, such as ghrelin and somatostatin, thereby further linking microbial activity with the endocrine control of growth processes [13,110].

Conversely, disturbances to the microbiome associated with dysbiosis, a high-fat diet or low fibre intake may reduce SCFA production and disrupt GH/IGF-1 signalling pathways. This can contribute to early metabolic dysregulation and increased cardiovascular risk in adolescents [175,176]. Metabolic disorders such as obesity and undernutrition frequently occur alongside alterations in the GH/IGF-1 axis and concurrent dysbiosis, highlighting the bidirectional nature of these interactions. This relationship is particularly critical during adolescence, when both hormonal systems and microbial communities undergo rapid maturation [177,178]. These findings provide a strong rationale for developing dietary and precision fermentation-based interventions that modulate the gut microbiome. Such strategies could support the optimal functioning of the GH/IGF-1 axis, improve metabolic outcomes and ultimately reduce early cardiometabolic risk in adolescent populations.

### 4.6. Microbiome Interactions with Leptin and Neuroendocrine Appetite Regulation

Clinical observations suggest that leptin, primarily secreted by adipocytes, plays a key role in regulating satiety, energy balance and immune function [179]. There is emerging evidence that the gut microbiota can indirectly modulate circulating leptin levels via metabolic pathways, particularly through the production of SCFAs. These metabolites interact with immune signalling, lipid metabolism, and neuroendocrine circuits, thereby influencing host metabolic regulation [177,180]. SCFAs, including acetate, propionate, and butyrate, stimulate enteroendocrine cells and hypothalamic pathways, which affect leptin sensitivity and the signalling networks that govern appetite, energy expenditure, and adiposity [59]. Acting as metabolic messengers, SCFAs link the gut ecosystem with endocrine and central nervous system pathways that regulate energy balance and feeding behaviour [117]. This regulatory network is particularly important during adolescence, a period characterised by the neuroendocrine system’s heightened sensitivity to dietary, microbial, and hormonal influences.

While evidence indicates that dysbiosis of the gut microbiota disrupts key regulatory mechanisms, shifts in microbial composition increase intestinal permeability and promote metabolic endotoxemia, thereby driving chronic low-grade inflammation, and experimental studies have demonstrated that elevated circulating levels of LPS and other pro-inflammatory mediators can impair leptin receptor signalling in the hypothalamus, ultimately leading to leptin resistance [129,181]. This inflammatory state interferes with the normal secretion and signalling of key metabolic hormones, including leptin and insulin [182]. This promotes insulin resistance, impaired satiety and dysregulated energy homeostasis—processes strongly associated with the development of metabolic and cardiovascular disorders [181,183]. Furthermore, adolescents who consume diets high in ultra-processed foods and low in dietary fibre or saturated fats are particularly susceptible to leptin resistance associated with dysbiosis, highlighting the close interplay between nutrition, microbial composition, and neuroendocrine regulation [184].

It is increasingly recognised that these findings emphasise the importance of maintaining balanced gut microbiota during adolescence to ensure optimal metabolic and hormonal regulation and to prevent early cardiometabolic disturbances, particularly as precision-fermented functional ingredients—including SCFA-enhancing prebiotics and postbiotic metabolites—offer a promising strategy to restore leptin sensitivity, attenuate low-grade inflammation and support proper neuroendocrine control of appetite in adolescent populations [126,174,185].

### 4.7. Integrated Microbiome–Hormone–Metabolism Interactions in Adolescent Cardiovascular Risk

A growing body of evidence shows that this microbially mediated regulation does not operate in isolation, but forms part of a broader network of interactions between the gut microbiome, endocrine signalling and metabolic pathways, providing a conceptual bridge to understanding how microbial metabolites such as SCFAs and TMAO influence the secretion and activity of key metabolic hormones—including insulin, GLP-1, PYY, ghrelin and leptin—alongside sex steroids and the GH/IGF-1 axis, thereby shaping appetite regulation, glucose and lipid metabolism and overall energy homeostasis during adolescence [1,186,187,188,189].

Microbiome-derived metabolites play a central role in the processes involved. SCFAs support metabolic homeostasis by modulating immune responses, enhancing intestinal barrier integrity and regulating lipid and glucose metabolism. This promotes insulin sensitivity and metabolic flexibility [104]. In contrast, TMAO—produced from dietary precursors such as choline, L-carnitine, and phosphatidylcholine-has been linked to endothelial dysfunction and pro-atherogenic signalling. Elevated TMAO levels contribute to vascular inflammation, reducing nitric oxide bioavailability and activating NF-κB and PKC pathways, thereby accelerating atherosclerotic processes. These properties position TMAO as a potential early biomarker of cardiometabolic risk in adolescents [135,190]. These findings highlight the importance of maintaining microbial balance during adolescence, a period characterised by increased metabolic plasticity and dynamic hormonal reorganization.

In parallel, PF technologies have enabled the development of novel functional ingredients, including lipids and sterols that are well characterised for their cardioprotective properties. Engineered microbial systems can produce long-chain omega-3 fatty acids, such as eicosapentaenoic acid (EPA) and docosahexaenoic acid (DHA), as well as structured lipids and phytosterols, which have traditionally been sourced from marine or plant-based materials [191,192]. EPA and DHA reduce hepatic triglyceride synthesis by downregulating enzymes involved in de novo lipogenesis, while simultaneously enhancing β-oxidation and stabilising cardiomyocyte membranes [193]. They also shift eicosanoid production towards less pro-inflammatory mediators, thereby reducing vascular inflammation and improving endothelial function [194]. Precision-fermented phytosterols further contribute to cardiometabolic protection by competing with dietary cholesterol for intestinal absorption, thereby increasing faecal sterol excretion and lowering circulating LDL cholesterol levels [195,196]. These PF-derived compounds counteract dyslipidaemia and pro-inflammatory lipid signalling associated with Western dietary patterns, offering a targeted, sustainable strategy to support vascular health in adolescents.

Furthermore, changes to diet and the composition of gut microbes are now seen as effective ways to boost metabolic stability during adolescence [197,198,199]. Studies have shown that fibre-rich diets, supported by selected prebiotic and probiotic approaches, can reduce systemic inflammatory activity and influence key hormonal pathways involving leptin, ghrelin and insulin. Early abnormalities in blood pressure, lipid profiles and glucose handling are countered by these changes, thereby lowering cardiometabolic risk in young individuals [197,198,199]. During puberty, there are dynamic changes in insulin-like growth factor 1 (IGF-1) levels, sex steroid profiles, and metabolic hormone regulation, which occur in parallel with shifts in gut microbiota composition. This makes the pubertal period particularly susceptible to microbial influences [200,201]. Consequently, dysbiosis during adolescence may lead to the premature onset of cardiometabolic dysfunction.

Emerging data suggests that integrating precision-fermented functional ingredients into adolescent diets may modulate the interactions between the microbiome, hormones and metabolism synergistically [202,203]. These approaches provide a scientifically based method to improve metabolic regulation, support hormonal balance, and ultimately reduce early cardiometabolic risk [1].

## 5. Epidemiology of Early Cardiovascular Risk in Polish Adolescents in an International Context

### 5.1. Early Cardiovascular Risk in Polish Adolescents

The epidemiological profile of Polish adolescents is crucial for understanding the biological and nutritional pathways outlined in earlier sections. It demonstrates how the described interactions between diet, microbiome and hormones manifest as measurable cardiometabolic vulnerability in real-world conditions. Cardiovascular diseases remain the leading cause of mortality among adults in Poland and globally [204], yet accumulating evidence shows that their biological roots frequently appear much earlier, during childhood and adolescence, underscoring the need for timely detection and preventive action. National and regional epidemiological data consistently reveal a high and rising prevalence of modifiable cardiovascular risk factors in Polish young people, including excess body weight, abdominal adiposity, elevated blood pressure, and lifestyle patterns that reinforce metabolic strain [205,206]. Current estimates suggest that 18–24% of school-aged adolescents are overweight or obese based on BMI criteria, and this figure may rise to 26% when body fat percentage is considered [207]. These figures put Poland above the levels usually seen in Western Europe (14–20%) and closer to those in Central and Southern Europe, where adolescent obesity rates are among the highest in Europe.

Excess adiposity and increased waist circumference in children and adolescents are strongly associated with elevated cardiovascular risk markers, including dyslipidaemia and hypertension [208]. While many Polish studies focus on obesity prevention in young people, relatively few address long-term cardiovascular outcomes in this population. Nevertheless, consistent international evidence shows that excess body weight in early life often persists into adulthood, substantially increasing the risk of cardiovascular disease later in life [209,210]. This places Poland within a broader global trend whereby early-life adiposity predicts future cardiometabolic burden. These epidemiological patterns emphasise the importance of examining the diet–microbiome–hormone pathways in the Polish adolescent population, given the high prevalence of modifiable risk factors during this developmental period.

The underlying pathophysiological mechanisms include insulin resistance, which contributes to renal sodium retention and increased sympathetic nervous system activity, thereby promoting hypertension [211]. At a molecular level, hyperinsulinaemia impairs endothelial function by reducing the bioavailability of nitric oxide (NO) through the downregulation of endothelial nitric oxide synthase (eNOS), as well as by promoting endothelial cell apoptosis. This ultimately leads to a reduced vasodilatory capacity and early vascular dysfunction [212,213]. Dyslipidaemia, characterised by elevated triglycerides and LDL cholesterol and reduced HDL cholesterol, is another key risk factor frequently observed in overweight and obese young people [214]. Studies in Poland confirm significantly higher concentrations of total cholesterol, LDL cholesterol and triglycerides in obese children and adolescents compared to those of normal weight [215,216,217], consistent with findings from other European and North American populations [218,219].

Abdominal obesity, which is a particularly strong predictor of cardiometabolic complications, is commonly observed among Polish adolescents and often occurs alongside hypertension and dyslipidaemia. Baran et al. (2025) reported stage I-II hypertension in up to 23.6% of adolescents aged 6–17 years [207]. This is notably higher than the European average of 10–15%, and comparable to levels observed in the United States. Regional studies further support this pattern. In the Silesian region, for example, 14.1% of high school students were classified as overweight and 2% as obese. Their dietary habits were characterised by high saturated fat intake and low consumption of fruit and vegetables—factors strongly associated with increased cardiovascular risk [17]. Similarly, in the Małopolska region, adolescents identified smoking (36.5%), alcohol consumption (35.2%), and unhealthy dietary habits (34.1%) as significant risk factors for cardiovascular disease [220]. These values are comparable to or slightly higher than those reported in other European countries.

These national observations are consistent with global trends. According to the WHO (2025), overweight and obesity in children and adolescents are increasing rapidly worldwide [221]. In 2024, they affected around 35 million children under 5 years old and over 390 million individuals aged 5–19 years, including more than 160 million people living with obesity. The prevalence of obesity in this age group has quadrupled since 1990. In Poland, approximately one in four adolescents is affected, placing the country among those with the fastest-increasing rates of metabolic disorders in young people [222]. Notably, conditions typically associated with adulthood, such as hypertension, dyslipidaemia, insulin resistance and early vascular dysfunction, are increasingly being diagnosed during adolescence, suggesting an earlier accumulation of cardiovascular risk factors [223,224]. These findings highlight the need for comprehensive preventive strategies targeting diet, lifestyle and metabolic regulation during adolescence. Emerging evidence suggests that interventions capable of modulating gut microbiota composition may be an effective way of reducing early cardiometabolic risk alongside traditional lifestyle approaches [225,226].

### 5.2. Biological Mechanisms Underlying Early Cardiovascular Risk in Adolescence

The accumulation of cardiovascular risk factors during adolescence triggers a series of biological processes that accelerate vascular ageing and induce early atherosclerotic changes, frequently occurring prior to the manifestation of clinical symptoms [224,227]. Excess body weight, particularly abdominal obesity, is a key driver of cardiometabolic dysfunction. Visceral adipose tissue is metabolically active and releases pro-inflammatory cytokines, such as TNF-α and IL-6, as well as adipokines, including leptin and adiponectin. These mediators interact with the gut microbiome, further amplifying inflammatory signalling pathways [228,229]. Sustained low-grade inflammation contributes to endothelial dysfunction, reduced nitric oxide (NO) bioavailability and accelerated arterial stiffening [230]. In parallel, increased free fatty acid flux from visceral fat promotes hepatic overproduction of atherogenic lipoproteins, resulting in dyslipidaemia characterised by elevated LDL cholesterol and reduced HDL cholesterol [214]—patterns already documented in Polish youth [207].

In light of accumulating evidence, another key component of early cardiometabolic dysfunction is insulin resistance, a disturbance that emerges with striking frequency in obese adolescents and reveals how profoundly metabolic and hormonal systems can become dysregulated during this vulnerable developmental window. It is now well appreciated that chronic hyperinsulinaemia does far more than reflect impaired glucose handling: it actively drives pathophysiological change by intensifying sympathetic nervous system activity, increasing renal sodium retention and stimulating vascular smooth muscle proliferation—a triad of effects that, taken together, lays the physiological groundwork for the development of hypertension [231,232]. Epidemiological data indicate that around 20–25% of overweight or obese Polish adolescents have elevated blood pressure [207]. Emerging evidence also suggests that gut microbiota dysbiosis exacerbates insulin resistance by altering SCFA production, disrupting enteroendocrine signalling, and enhancing systemic inflammation [128]. Furthermore, studies such as those by Wieniawski and Werner (2022) identify BMI, waist-to-hip ratio and family history of hypertension as strong predictors of elevated blood pressure in young people, highlighting the interaction between genetic susceptibility and modifiable environmental factors [233].

Longitudinal evidence indicates that cardiovascular risk factors that emerge in childhood and adolescence tend to persist into adulthood. This phenomenon is known as “tracking” [234,235]. This persistence is largely driven by sustained lifestyle patterns, such as an increased consumption of highly processed foods and reduced physical activity. Epidemiological studies report rising rates of hypertension, dyslipidaemia and obesity in children and adolescents, suggesting an increasing burden of cardiovascular disease in later life [236]. Tracking reflects the biological continuity of early metabolic disturbances and highlights the stabilising role of diet–microbiome–hormone interactions in reinforcing long-term risk trajectories. Notably, elevated blood pressure, dyslipidaemia, insulin resistance and gut dysbiosis in young people are strongly associated with cardiovascular events in adulthood [237]. Cohort studies further confirm that adverse metabolic and vascular profiles in childhood predict increased cardiovascular morbidity later in life [238]. These findings suggest that Polish adolescents follow global patterns of early cardiometabolic risk development, exhibiting similar or slightly more pronounced vascular alterations compared to their Western European peers.

Structural and functional cardiovascular changes can be detected as early as adolescence. Obese children exhibit reduced arterial elasticity, increased carotid intima-media thickness and impaired endothelial-dependent vasodilation; these are all early markers of atherosclerotic progression [239]. Left ventricular hypertrophy (LVH), another early sign of cardiovascular strain, has also been observed in obese paediatric patients and is closely linked to hyperinsulinaemia and an increased cardiac workload [240,241]. Recent studies further indicate that microbial metabolites, such as SCFAs and TMAO, influence endothelial function, lipid metabolism and vascular inflammation in adolescents. This links gut microbiota composition directly to early cardiovascular remodelling. These findings are consistent with international evidence demonstrating that early vascular changes are measurable and predictive of adult cardiovascular disease outcomes [242,243]. These processes demonstrate how metabolic, inflammatory, and haemodynamic pathways converge during adolescence, accelerating the accumulation of cardiovascular risk.

In light of robust epidemiological and behavioural evidence, it is now clear that behavioural and dietary factors are fundamental to adolescent cardiometabolic health. Low levels of physical activity, poor dietary quality, and high consumption of ultra-processed foods create a metabolic environment that markedly increases cardiovascular risk [244]. Concurrently, long-term observational studies consistently demonstrate that adolescents who adhere to healthier dietary patterns exhibit more favourable metabolic profiles and a lower incidence of early cardiometabolic disturbances. This highlights the long-lasting impact of lifestyle behaviours established during this developmental period [245]. Furthermore, international cohort analyses compellingly demonstrate that adolescents with greater nutritional knowledge are at substantially lower risk of cardiovascular disease in early adulthood, highlighting the protective role of dietary competence in shaping long-term cardiometabolic health [246]. In contrast, a high intake of ultra-processed foods has been associated with poorer lipid profiles, increased adiposity and a higher BMI [234,247]. Furthermore, emerging research suggests that modulating the gut microbiome through diet may represent an additional strategy for reducing early cardiovascular risk by enhancing SCFA production, improving hormonal signalling and reducing systemic inflammation [248]. These global trends are also evident in Polish adolescents, whose dietary behaviours are increasingly similar to Western patterns characterised by the consumption of high-calorie, nutrient-poor foods [249].

As the pathological processes underlying cardiovascular disease begin early in life, it is essential to identify and modify risk factors in a timely manner in order to prevent long-term cardiovascular morbidity. Preventive strategies include improving diet, increasing physical activity, providing health education and carrying out targeted interventions to improve metabolic health and reduce adiposity [250]. In this context, precision nutrition approaches, including precision-fermented functional ingredients, offer promising tools for modulating diet–microbiome–hormone interactions and supporting cardiometabolic health in adolescents [251,252]. Such strategies are particularly relevant in Poland, where 20–25% of adolescents are overweight or obese, and hypertension, dyslipidaemia and insulin resistance are becoming more prevalent.

In summary, early-life adiposity and metabolic disturbances in Polish adolescents reflect global trends and arise from interconnected molecular, behavioural, and environmental mechanisms. Integrating dietary interventions with microbiome-targeted strategies could provide an informed approach to reducing early cardiometabolic risk and improving long-term cardiovascular outcomes. As these risk factors persist into adulthood and are strong predictors of future cardiovascular disease, early prevention in adolescence is crucial for altering lifelong cardiometabolic trajectories.

## 6. Microbial Metabolites, Inflammatory Pathways, and Metabolic Dysregulation in Adolescents

Gut-derived metabolites significantly influence metabolic and immunological pathways in the host, thereby shaping susceptibility to obesity, insulin resistance and early cardiovascular risk [1,253]. Medium-chain (MCFAs) and SCFAs fatty acids act as signalling molecules via receptors such as GPR41 and GPR43 in adipocytes and immune cells, which improves lipid metabolism, modulates enteroendocrine hormone secretion and reduces insulin resistance [254]. These receptor-mediated mechanisms enable the gut microbiome to regulate systemic metabolic homeostasis via nutrient-derived metabolites, thereby linking diet, microbial ecology, and endocrine signalling.

In addition to beneficial metabolites such as SCFAs, certain microbiota-derived compounds may have adverse effects on cardiometabolic health. TMAO, for example, is a pro-atherogenic microbial metabolite produced from dietary precursors rich in choline, L-carnitine and phospholipids, which are commonly found in red meat and eggs. Gut microbes convert these precursors to trimethylamine (TMA), which is then oxidised in the liver [255]. Elevated serum TMAO levels have been linked to accelerated atherogenesis, inflammatory cell activation, endothelial dysfunction, and early vascular remodelling [256]. In adolescents, high TMAO concentrations have been found to correlate with dyslipidaemia, impaired glucose homeostasis, and subclinical markers of cardiovascular risk [138], thereby positioning TMAO as a critical link between diet, microbial metabolism, and early cardiometabolic dysfunction.

### 6.1. Intestinal Barrier Integrity and Metabolic Endotoxemia

The gut microbiota plays a central role in maintaining the integrity of the intestinal barrier by preventing excessive permeability and limiting the translocation of microbial components [257]. Disruption to the microbiome can lead to dysbiosis, which compromises the integrity of tight junctions and increases intestinal permeability. Consequently, LPS and other microbial fragments can enter the bloodstream, triggering low-grade inflammation—a condition known as metabolic endotoxemia [143]. LPS activates the NF-κB signalling pathway, thereby increasing the production of pro-inflammatory cytokines and promoting insulin resistance, endothelial dysfunction and the formation of atherosclerotic lesions [258]. These mechanisms provide a link between gut microbial imbalance, metabolic syndrome and early cardiovascular risk in adolescents.

At the compositional level, dysbiosis is characterised by reduced microbial diversity and altered proportions of dominant bacterial taxa, including shifts in the *Firmicutes*: *Bacteroidetes* ratio [259]. These alterations are associated with impaired energy metabolism, lipid dysregulation, glucose intolerance and chronic inflammation [257]. Chronic exposure to Western-style diets exacerbates these disturbances further by increasing the production of pro-inflammatory microbial metabolites and reducing the abundance of taxa that produce SCFAs, thereby amplifying systemic inflammation and metabolic dysfunction [260]. Therefore, dietary patterns and microbiome composition act synergistically to drive metabolic derangements.

Against the backdrop of rapidly advancing microbiome science, it is increasingly evident that the gut microbiome, beyond its structural and compositional roles, performs a wide spectrum of metabolic processes that exert profound effects on host physiology, particularly through its involvement in the metabolism of bile acids, vitamins, dietary bioactives and host-derived compounds, all of which collectively regulate lipid absorption, energy homeostasis and endocrine signalling [41]. Accumulating evidence suggests that individuals harbouring microbial configurations optimised for enhanced energy extraction may be at a higher risk of excessive weight gain and subsequent metabolic disturbances, highlighting the complex relationship between diet, microbial metabolism and host physiology [261]. Furthermore, consistent findings from experimental and clinical studies demonstrate that gut microbes can contribute to obesity by increasing caloric extraction from the diet, modulating host genes involved in fat storage, and shaping inflammatory pathways that reinforce metabolic dysfunction [262]. Although it remains challenging to define a single, universally applicable obesity-associated microbial signature, due to substantial interindividual variability and methodological differences across studies, several investigations have nonetheless reported an increased *Firmicutes*-to-*Bacteroidetes* ratio in individuals with obesity, suggesting a recurring—though not universally observed—microbial pattern linked to altered energy balance [259], even as other analyses caution that such associations may be inconsistent or context-dependent [263,264].

Importantly, these microbial and metabolic alterations are further shaped by dietary components and host immune responses [265]. For example, nutritional factors such as cholesterol, saturated fats, and simple sugars can promote inflammation [266], while microbial-associated molecular patterns, including LPS and peptidoglycan, can activate host immune pathways [267]. The dynamic interaction between dietary inputs, microbial metabolites, and immune signalling within the gut ecosystem highlights how environmental and biological factors converge to affect metabolic homeostasis and early cardiovascular risk in adolescents [1].

### 6.2. Microbial Regulation of Bile Acids, Vitamins, and Host-Derived Metabolites

Against the backdrop of rapidly expanding knowledge on host–microbe metabolic crosstalk, it is now well established that the gut microbiome plays a central role in metabolising bile acids, vitamins, dietary bioactives and host-derived compounds, thereby shaping multiple dimensions of host physiology [41]. Through the catalytic activity of specialised microbial enzymes, primary bile acids undergo deconjugation and biotransformation into secondary bile acids, which function as potent signalling molecules acting via receptors such as the farnesoid X receptor (FXR) and the Takeda G-protein-coupled receptor 5 (TGR5), creating a direct biochemical interface between microbial metabolism and host endocrine pathways [268]. These receptor-mediated interactions exert far-reaching metabolic consequences, as FXR- and TGR5-dependent signalling modulates lipid metabolism, glucose homeostasis and energy expenditure, thereby integrating microbial bile acid transformations with systemic metabolic regulation [269]. Therefore, this intricate metabolic dialogue contributes to controlling lipid absorption, systemic energy balance and endocrine signalling, highlighting the extent to which microbial activity influences host metabolic trajectories [41]. Accumulating evidence in this context suggests that individuals harbouring microbial profiles optimised for enhanced energy extraction may be more susceptible to weight gain and associated metabolic disturbances, thereby highlighting the metabolic implications of microbially mediated energy harvesting [262].

In addition to bile acid metabolism, the gut microbiota contributes to host metabolic regulation through multiple complementary mechanisms. For example, gut microbes can influence obesity by enhancing the extraction of calories from otherwise indigestible polysaccharides, regulating host fat storage genes, modulating bile acid signalling pathways and stimulating inflammatory responses that contribute to insulin resistance and metabolic dysregulation [270]. Furthermore, microbial communities are involved in the biosynthesis of essential vitamins, including vitamins K and B, which are involved in metabolic pathways relevant to energy metabolism and vascular function [271]. Alterations in these microbial metabolic activities have been associated with dyslipidaemia, impaired glucose tolerance and chronic low-grade inflammation—key features of early cardiometabolic risk [272].

The above-described mechanisms—including MCFA/SCFA signalling, TMAO-mediated atherogenesis, LPS-driven NF-κB activation, bile acid modulation and microbial regulation of energy extraction—highlight a network of interconnected pathways that link the gut microbiome to metabolic homeostasis in the host. These pathways also represent precise biochemical targets for next-generation bioactive compounds produced via precision fermentation. Such compounds can be designed to reduce TMAO formation by modulating microbial choline metabolism, enhance SCFA production or deliver SCFA analogues with improved bioavailability, strengthen intestinal barrier integrity through postbiotic peptides or butyrate-enhancing molecules, suppress NOD1- and NF-κB-mediated inflammatory signalling, modulate bile acid pools to improve lipid and glucose metabolism, shift microbial composition towards anti-inflammatory, metabolically favourable taxa. Precision fermentation technologies enable the controlled biosynthesis of such functional ingredients using engineered microorganisms. This offers a sustainable and scalable alternative to conventional food-derived bioactives, allowing targeted modulation of microbiome-mediated metabolic pathways [273,274,275].

These targeted strategies may be particularly relevant during adolescence, a period characterised by profound metabolic and hormonal remodelling. During puberty, the dynamic interactions between the gut microbiome, endocrine signalling (including insulin, leptin and the GH/IGF-1 axis) and dietary exposure create a period of metabolic plasticity, during which early interventions can have long-term effects on cardiometabolic health [1]. Consequently, functional ingredients that modulate the microbiome, derived from precision fermentation, may represent a promising approach to reducing early cardiometabolic risk and supporting optimal metabolic development in adolescents [276].

## 7. Dietary Modulation of the Microbiome as a Cardiometabolic Regulator in Adolescents

Diet is one of the most powerful factors that influence the composition, diversity and metabolic activity of the gut microbiome. Its impact is particularly pronounced during adolescence, a period of development characterised by heightened metabolic plasticity and the early establishment of cardiovascular risk profiles [1]. Plant-based and Mediterranean dietary patterns consistently promote microbial communities enriched in fibre-fermenting taxa, leading to increased production of SCFAs [277]. SCFAs act via receptors such as GPR41 and GPR43 to enhance insulin sensitivity, regulate lipid metabolism and stimulate anorexigenic hormones, including GLP-1 and PYY [90]. Through these receptor-mediated mechanisms, SCFAs influence hepatic glucose production, adipose tissue metabolism and inflammatory signalling pathways, thereby contributing to improved metabolic homeostasis and reduced cardiometabolic risk [278]. Importantly, these molecular effects counteract the early stages of atherogenesis and metabolic dysfunction, emphasising the importance of diet–microbiome interactions in determining adolescent cardiometabolic health.

Consistent with these observations, comparative studies demonstrate that Mediterranean-style dietary patterns are more effective than Western diets in shaping a cardioprotective microbiome. Studies by Beam et al. (2021) [279] and Buil-Cosiales et al. (2016) [280] suggest that diets containing high levels of fruit, vegetables, whole grains, legumes, nuts and fish can increase microbial diversity and SCFA production, while reducing circulating TMAO levels. In contrast, diets high in saturated fats and ultra-processed foods promote dysbiosis by decreasing SCFA-producing bacteria and increasing Gram-negative taxa that release LPS. This shift activates TLR4-NF-κB-mediated inflammatory pathways, which are strongly associated with insulin resistance and vascular injury [277]. These findings show how changes in the gut microbiome caused by diet can quickly affect inflammatory responses, lipid metabolism, and endothelial function, thus influencing the early development of cardiovascular risk in adolescents. These contrasting dietary effects further highlight the importance of the microbiome as a central link between nutrition and cardiometabolic health [1].

### 7.1. Short-Term Dietary Interventions Reveal the Microbiome’s Rapid Responsiveness

Short-term dietary interventions provide compelling evidence of the dynamic responsiveness of the gut microbiome to nutritional exposures. For instance, Bourdeau-Julien et al. (2023) [281] showed that high-fat/high-sugar diets can modify the composition of the microbiome within days, whereas following a Mediterranean diet can decrease harmful metabolites within 48 h. Similarly, Rejeski et al. (2022) [282] reported rapid shifts in microbial diversity and strain abundance after adopting a Mediterranean dietary pattern. However, Patloka et al. (2024) [283] noted that these changes may be reversible when previous dietary habits are resumed. Taken together, these findings suggest that the adolescent microbiome is highly adaptable—both susceptible to detrimental dietary habits and responsive to nutritional improvement.

Importantly, the molecular consequences of dietary modulation extend beyond taxonomic shifts in microbial communities to functional metabolic outputs. Undigested proteins and fats can serve as substrates for microbial fermentation, leading to the production of either beneficial SCFAs or potentially harmful metabolites, such as ammonia, phenols and branched-chain fatty acids [55]. Polyphenols and flavonoids, which are abundant in plant-based diets, modulate microbial gene expression, enhance intestinal barrier integrity and suppress inflammatory signalling [284]. These compounds also act as prebiotic-like substrates, selectively promoting the growth of beneficial taxa such as *Bifidobacterium* and *Lactobacillus* while inhibiting pro-inflammatory microorganisms [285]. These observations suggest that targeted dietary strategies, and potentially precision-fermented bioactive compounds, could be used to replicate or amplify the beneficial effects of the microbiome in preventing cardiometabolic disorders in adolescents.

These diet–microbiome interactions are further supported by links between dysbiosis and the development of chronic diseases. Dysbiosis has been associated with obesity, insulin resistance, non-alcoholic fatty liver disease (NAFLD), inflammatory bowel disease, allergies and mental health disorders [286]. Obesity-associated taxa, such as Enterobacteriaceae and Desulfovibrionaceae, produce LPS, which activates TLR4 via MyD88-dependent and -independent pathways [287]. Qin et al. (2021) [288] demonstrated that inhibiting the TLR4/TRIF-MyD88-independent pathway can prevent insulin resistance induced by a high-fat diet, highlighting a precise molecular target for intervention. These findings emphasise that modulating microbial signalling pathways through diet is a promising strategy for mitigating inflammation-driven metabolic dysfunction during adolescence [1].

### 7.2. Dietary Patterns High in Fibre and SCFA-Producing Microbiota

Improved glucose control, lipid handling and immune function are all linked to diets that focus on plant-based foods [289,290]. Such diets promote a more diverse and stable gut microbiome, especially when red meat, refined sugars and ultra-processed products are limited [289]. One key factor in these benefits is the increased availability of fermentable fibre, which promotes the growth of fibre-utilising bacteria and enhances the production of SCFAs with metabolic and anti-inflammatory properties [63]. These SCFAs—acetate, propionate and butyrate—provide energy for colonocytes, influence hepatic gluconeogenesis and modulate immune signalling. This strengthens the intestinal barrier and supports systemic metabolic homeostasis [73].

These findings are in line with the idea that plant-based dietary patterns are good for your gut bacteria and can help control your blood sugar levels. Two examples of plant-based diets that have been shown to have a positive effect on your gut bacteria and blood sugar levels are the Mediterranean diet and the DASH (Dietary Approaches to Stop Hypertension) diet. The DASH diet is designed to reduce blood pressure and is characterised by high intakes of fruits, vegetables, whole grains and low-fat dairy products with limited sodium and saturated fat. [291]. These dietary patterns enhance SCFA production and reduce circulating TMAO levels, a metabolite associated with an increased risk of cardiovascular and metabolic diseases, while also promoting greater microbial diversity, which protects against a wide range of non-communicable diseases [292]. Experimental studies in rodents, non-human primates and humans demonstrate that the Mediterranean diet reshapes the gut microbiome through its high fibre and polyphenol content, thereby stimulating the growth of beneficial bacteria and increasing SCFA production [293,294,295].

**Figure 4:**
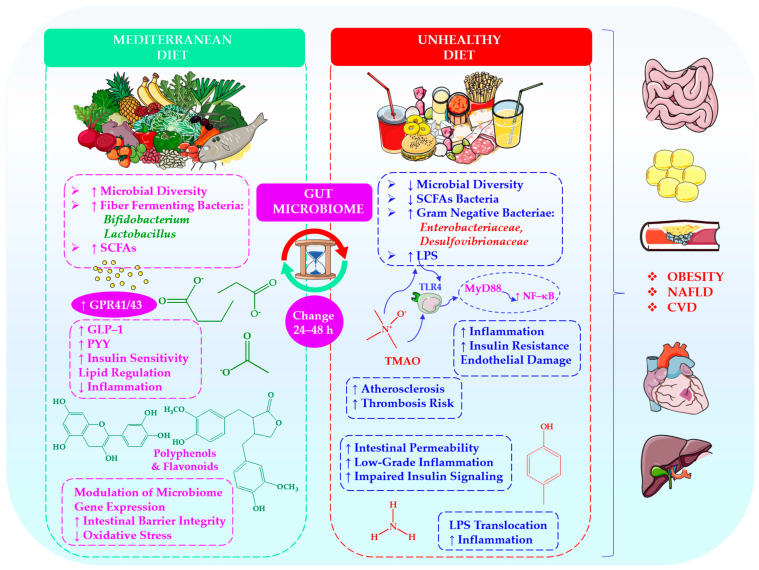
Diet–microbiome interactions in adolescence shape cardiometabolic risk. Dietary patterns are major modulators of gut microbiome composition, diversity, and metabolic activity, particularly during adolescence, a period of increased metabolic plasticity and early cardiometabolic risk programming. Plant-based and Mediterranean diets promote fiber-fermenting bacteria (e.g., *Bifidobacterium*, *Lactobacillus*) and increase the production of short-chain fatty acids (SCFAs), which activate GPR41 and GPR43 receptors, enhance insulin sensitivity, regulate lipid metabolism, and stimulate anorexigenic hormones (GLP-1, PYY). These effects contribute to improved metabolic homeostasis and reduced cardiometabolic risk. In contrast, Western diets rich in saturated fats and ultra-processed foods induce dysbiosis, characterized by reduced SCFA-producing bacteria and increased Gram-negative taxa producing lipopolysaccharide (LPS). This activates pro-inflammatory TLR4–NF-κB signaling, promoting insulin resistance and endothelial dysfunction. Additionally, microbial metabolites such as TMAO, ammonia, and phenols further exacerbate inflammation, impair gut barrier integrity, and contribute to atherogenesis. Polyphenols and flavonoids further support microbial balance, enhance gut barrier function, and suppress oxidative stress and inflammation. Image provided by Servier Medical Art (https://smart.servier.com, 6 April 2026), licensed under CC BY 4.0 (https://creativecommons.org/licenses/by/4.0/, accessed on 9 April 2026). Abbreviations: CVD—cardiovascular disease; GLP-1—glucagon-like peptide-1; LPS—lipopolysaccharide; MyD88—myeloid differentiation primary response 88; NAFLD—non-alcoholic fatty liver disease; NF-κB—nuclear factor kappa-light-chain-enhancer of activated B cells; PYY—peptide YY; SCFAs—short-chain fatty acids; TLR4—toll-like receptor 4; TMAO—trimethylamine N-oxide.

Figure 4 illustrates how dietary patterns during adolescence influence the composition of the gut microbiome and metabolic activity, thereby impacting cardiometabolic risk. It contrasts the beneficial effects of plant-based diets with the detrimental impact of Western diets on inflammation, insulin resistance and vascular health.

Notably, these diet-induced microbiome changes could have a significant impact during adolescence, a developmental period characterised by the ongoing maturation of the gut microbiota, endocrine signalling and metabolic regulation. This makes it a critical time for targeted nutritional interventions [1].

### 7.3. Microbial Metabolites, Nitric Oxide Pathways and Precision-Fermented Targets for Adolescent Cardiovascular Health

Microbial metabolites are key mediators that link diet, microbiome composition and cardiovascular risk. SCFAs promote metabolic flexibility and anti-inflammatory signalling, whereas TMAO and LPS contribute to endothelial dysfunction and systemic inflammation [296]. In addition to their metabolic effects, SCFAs regulate host energy balance, modulate immune responses and influence the secretion of enteroendocrine hormones involved in appetite and glucose regulation, thereby supporting systemic cardiometabolic homeostasis [74]. Given these multifaceted roles, microbiome-derived metabolites are attractive therapeutic targets.

Precision fermentation offers the possibility of engineering bioactive compounds that can selectively modulate these pathways with greater specificity than diet alone. For instance, precision-fermented SCFA analogues could activate GPR41/GPR43 signalling independently of high fibre intake [90], while engineered inhibitors of microbial cutC/D enzymes could suppress trimethylamine (TMA) production at its microbial origin, thereby reducing TMAO-driven atherogenesis [297,298]. These approaches demonstrate the potential of precision fermentation to target microbiome-dependent metabolic pathways involved in the early stages of cardiometabolic dysfunction in adolescents [126,299].

In parallel, the nitrate–nitrite–nitric oxide pathway is another microbiome-dependent mechanism with direct cardiovascular relevance. Dietary nitrates, derived from leafy vegetables, increase the availability of nitrates in the body. Oral bacteria then convert these nitrates into nitrites, which can be reduced to NO under hypoxic conditions [300,301,302]. NO plays a central role in regulating vascular tone, mitochondrial respiration, neurotransmission and muscle function [303]. Consuming nitrate-rich foods such as beetroot juice has been shown to improve endothelial function, lower blood pressure and enhance exercise performance. Skeletal muscle acts as an important nitrate reservoir [304]. Furthermore, NO is a key regulator of endothelial health, mediating vasodilation, inhibiting platelet aggregation and limiting vascular inflammation, thereby protecting against the onset of atherosclerosis [305]. However, the efficiency of this pathway depends heavily on the presence of nitrate-reducing oral bacteria, which can be depleted by Western dietary patterns, the excessive use of antiseptic mouthwashes and microbial dysbiosis [306,307]. In this context, precision-fermented NO-enhancing postbiotics may help to overcome these limitations by providing stable nitrite-generating compounds that are independent of microbial variability.

In addition to therapeutic targeting, the value of microbiome-derived biomarkers as diagnostic and prognostic tools in cardiometabolic health is increasingly recognised. For example, Hajjo et al. (2022) [42] highlighted the potential of microbial gene and metabolite profiles for the early identification of metabolic risk, and Wu et al. (2024) [308] proposed the concept of a ‘core microbiome signature’ as a comprehensive health status indicator. Advances in multi-omics technologies, including metagenomics, metabolomics and transcriptomics, now enable the identification of microbial metabolic pathways associated with early cardiometabolic disturbances. These developments highlight the importance of interventions that can precisely modulate microbial pathways, rather than relying solely on broad dietary modifications [309,310]. Precision fermentation allows compounds to be designed that target specific microbial enzymes, metabolic pathways or host receptors, offering a level of precision that cannot be achieved through diet alone [27].

Accordingly, fermented products have a wide range of potential applications in the prevention of cardiometabolic diseases. These include SCFA-enhancing postbiotics that activate GPR41/GPR43 signalling and improve insulin sensitivity, TMA/TMAO pathway inhibitors that block microbial choline metabolism and reduce endothelial inflammation, NO-enhancing molecules that support vascular function in adolescents with early cardiometabolic risk, TLR4 pathway modulators that suppress LPS-induced inflammation, designer prebiotic oligosaccharides that selectively promote cardioprotective taxa, polyphenol-derived fermentation products that stabilise barrier integrity and reduce oxidative stress [311,312,313]. Importantly, precision fermentation technologies enable the scalable production of these targeted bioactive compounds using engineered microbial systems, providing a sustainable alternative to the conventional extraction of such compounds from plant or animal sources [27,314]. Precision-fermented compounds represent a promising strategy for reducing early cardiovascular risk and supporting metabolic and hormonal health during adolescence by targeting diet-responsive molecular pathways with greater specificity, stability, and scalability [315].

### 7.4. Implications for Precision-Fermented Bioactive Compounds

Consistent modulation of the gut microbiome through plant-rich dietary patterns highlights several potential targets for replication or amplification via precision fermentation. These include SCFA-enhancing compounds that mimic the metabolic benefits of high-fibre diets, designer oligosaccharides that selectively promote beneficial taxa associated with Mediterranean-like microbial profiles, polyphenol-derived postbiotics that are produced through fermentation to mimic the effects of plant bioactives on the microbiome [103,285,316]. Additionally, compounds that suppress TMA/TMAO formation can mimic the cardioprotective effects of plant-based diets [317]. Further targets include microbial enzyme inhibitors that regulate bile acid metabolism, bioactive peptides that strengthen the integrity of the intestinal barrier, and postbiotic metabolites that modulate inflammatory signalling pathways, including NF-κB and TLR4 [318,319].

Beyond these specific targets, precision-engineered microbial metabolites that stabilise microbial diversity and support systemic metabolic homeostasis represent an additional, complementary strategy [319]. These approaches are particularly relevant for adolescents, whose dietary patterns often deviate from recommended nutritional guidelines, and whose microbiome is highly responsive to targeted interventions. Moreover, adolescence is characterised by rapid hormonal, metabolic and microbiome-related changes, suggesting that interventions modulating the interplay between diet, microbial composition and endocrine signalling could have long-lasting effects on cardiometabolic health [1,25,87]. These considerations support the concept that precision-fermented functional ingredients provide a promising and scalable framework for developing targeted nutritional strategies [320]. Such approaches may help to prevent early cardiovascular risk and promote optimal metabolic development in adolescents.

## 8. Sustainable Development Framework for Adolescent Cardiovascular Health in the Era of Precision Fermentation

Adopted by the United Nations in 2015, the 2030 Agenda for Sustainable Development and the Sustainable Development Goals (SDGs) provide a global framework for enhancing the well-being of both people and the planet. SDG 3 focuses on health and well-being, emphasising disease prevention throughout life, including during adolescence—a critical period when behavioural and metabolic risk factors for non-communicable diseases (NCDs), particularly cardiovascular diseases (CVDs), begin to emerge [321]. SDG Target 3.4 aims to reduce premature mortality from NCDs by one third by 2030 through prevention, treatment and promoting mental health. As CVD risk is strongly influenced by diet, physical activity, tobacco use, sleep hygiene and other environmental factors established in early life, effective prevention strategies must begin during adolescence [322,323,324]. In addition to SDG 3, other goals, such as SDG 2 (Zero Hunger), SDG 12 (Responsible Consumption and Production) and SDG 13 (Climate Action), are closely linked to food systems and nutrition policies that influence cardiometabolic health outcomes [325,326,327]. This integrated approach is consistent with the growing potential of precision fermentation, a biotechnology that can produce specific functional ingredients to influence interactions between diet, the microbiome, and endocrine signalling—key factors in the development of early cardiometabolic risk [30,328,329].

Globally, CVDs account for around 32% of all deaths, with almost half of these being considered preventable through primary prevention [321]. There is increasing evidence that risk factors such as excess body weight, an unhealthy diet, physical inactivity and chronic stress originate in childhood and adolescence and persist into adulthood, thereby amplifying long-term cardiovascular risk [330]. Data from the WHO and the Health Behaviour in School-Aged Children (HBSC) study indicate that up to 80% of adolescents aged 11–17 do not engage in sufficient physical activity, while the consumption of ultra-processed foods and simple sugars is contributing to the increasing prevalence of obesity and hypertension [331,332]. Meanwhile, global dietary shifts towards highly processed, energy-dense foods are altering the composition of the gut microbiome and promoting metabolic dysregulation, thereby linking modern food environments to the growing burden of cardiometabolic disease in young populations. These trends reflect individual behavioural choices and structural constraints within contemporary food systems, highlighting the importance of interventions that integrate health promotion with sustainable food innovation during adolescence [53].

According to the UNDP (2025) [323], achieving sustainable health development requires long-term investment in supportive living environments that promote healthy dietary habits and physical activity from an early age. The WHO emphasises that plant-centred dietary patterns that are low in saturated fats, salt, and added sugars are essential for preventing obesity, hypertension, and type 2 diabetes [331]. By mitigating metabolic disturbances driven by ultra-processed diets frequently consumed during adolescence, these compounds can influence SCFA profiles, bile acid turnover, endothelial nitric oxide generation and insulin sensitivity [333,334,335]. Through these mechanisms, they enhance microbial diversity, stabilise metabolic signalling and reduce early cardiovascular risk. PF contributes to these effects by supplying high-purity functional ingredients with a low environmental footprint, including prebiotic fibres, postbiotics, bioactive peptides and microbially derived metabolites. Importantly, PF enables the scalable production of these bioactives using engineered microorganisms, offering a sustainable alternative to conventional agricultural extraction methods [27,336].

Such innovations are also consistent with the principles of sustainable food systems. For example, precision fermentation can reduce the environmental footprint of conventional agriculture by lowering greenhouse gas emissions, land use and water consumption [27,314,337]. In Poland, these priorities are reflected in national prevention strategies such as the National Health Programme 2021–2025, which incorporates nutrition education and school-based health promotion alongside measures aimed at reducing ultra-processed food consumption [338]. Structural interventions, including the taxation of sugar-sweetened beverages, restrictions on the marketing of ultra-processed foods to children and the development of infrastructure to encourage physical activity, can be further supported by incorporating plant-based and precision-fermented ingredients into diets, thereby improving nutrition without increasing the environmental burden [339]. Therefore, incorporating precision fermentation-derived functional ingredients into school meal programmes and public health nutrition policies could be an effective way to improve diet quality and advance sustainability goals simultaneously [340].

Both the WHO (2025) [321] and the UNDP (2025) [323] emphasise that integrated, cross-sectoral strategies can reduce CVD risk and promote health equity by improving access to nutritious and sustainable foods across socioeconomic groups. Countries that have implemented SDG-aligned initiatives, such as creating healthy school environments and adopting active urban planning strategies, have reported improvements in adolescent NCD indicators [341]. Health education programmes, such as the WHO-UNICEF Health Promoting Schools initiative, enhance adolescents’ knowledge of nutrition, physical activity and mental health. This leads to improved dietary behaviours, increased physical activity and a lower body mass index (BMI) [342,343]. Expanding these programmes to include biotechnology literacy and sustainability education could further empower adolescents to understand how innovations such as precision fermentation can support personal health and environmentally responsible food systems [27,314,337].

A coordinated approach that integrates health, environmental and economic policies is essential for achieving sustainable development. Promoting locally sourced, minimally processed foods and active lifestyles improves population health while reducing greenhouse gas emissions [323,344]. From an economic perspective, the early prevention of CVD reduces long-term healthcare costs and productivity losses. The WHO estimates that every USD 1 invested in NCD prevention yields up to USD 7 in long-term economic returns [321]. Monitoring systems such as the HBSC study (2023) [332] are crucial for tracking obesity, hypertension, dyslipidaemia and dietary patterns in adolescents, and could be expanded to evaluate the effect of precision fermentation-based interventions on cardiometabolic outcomes. Incorporating microbiome-based biomarkers and advanced dietary assessment tools into these systems could enhance the early detection of risk and the evaluation of targeted nutritional strategies even further.

In this context, precision fermentation emerges as a promising, sustainable approach to improving adolescent nutrition, modulating microbiome–endocrine interactions and reducing early cardiovascular risk, all the while supporting SDG targets. Integrating advances in biotechnology, nutrition science, and public health policy enables the development of precision fermentation-based innovations that offer a forward-looking framework for creating sustainable dietary strategies aimed at improving long-term cardiometabolic health outcomes in young populations [27,314].

## 9. Limitation

Although the current evidence on early cardiovascular risk in adolescents highlights well-characterised biological and behavioural pathways, there are several limitations that constrain the translation of these findings into sustainable, precision-targeted nutritional strategies. Most existing studies are observational, which restricts our ability to draw causal conclusions about the diet–microbiome–hormone axis and its role in early cardiometabolic dysfunction. Although cohort studies consistently demonstrate associations between dietary patterns, microbiome composition and cardiometabolic markers, they often lack the controlled experimental designs required to disentangle the complex interactions between host physiology [345,346,347], microbial ecosystems and dietary exposures. Additionally, research focusing on Polish adolescents is fragmented and lacks longitudinal data capturing the temporal interactions between diet, microbiome dynamics, and endocrine development. Furthermore, many studies rely on cross-sectional microbiome analyses, which fail to reflect adequately the highly dynamic nature of microbial ecosystems during adolescence, a period characterised by rapid physiological and hormonal changes [348,349].

Although precision fermentation is a promising area of nutritional biotechnology, it is still in its early stages of development. Few clinical trials have evaluated the use of precision-fermented functional ingredients in paediatric or adolescent populations [336,350], and further research is required to assess their long-term safety, metabolic effects and acceptability. Most of the available evidence comes from in vitro studies, animal models or adult cohorts, which limits the ability to directly extrapolate findings to adolescent physiology. Socioeconomic disparities in access to healthy diets and emerging food technologies may exacerbate health inequities further if precision fermentation-based solutions are not implemented within inclusive, affordable frameworks [351]. Furthermore, the regulatory frameworks governing novel fermented bioactive ingredients are still evolving in many regions, including the European Union. Comprehensive safety evaluations, including assessments of allergenicity, metabolic tolerance and long-term exposure, are therefore required prior to the widespread application of these ingredients in functional foods targeting adolescents [352].

Environmental considerations also represent an important area of ongoing uncertainty. Although preliminary life-cycle assessment (LCA) studies suggest that microbial fermentation platforms could reduce land use and greenhouse gas emissions compared to conventional animal-based food production methods, the full environmental impact is still not fully understood [353,354]. In particular, the energy demands associated with bioreactor operation, downstream processing and large-scale industrial fermentation must be carefully evaluated. These considerations emphasise the necessity of integrated, multidisciplinary research prior to the widespread implementation of precision fermentation technologies as a preventive strategy for cardiovascular disease in adolescents [352,353]. Consequently, future approaches must combine nutritional science, microbiome research, biotechnology, environmental assessment and public health policy to ensure that precision fermentation-based interventions are biologically effective, socially acceptable and environmentally sustainable.

## 10. Future Directions

Future research should prioritise longitudinal and studies that map the effects of precision-engineered nutrients on microbiome diversity, SCFA production, bile acid signalling and hormonal regulation during adolescence, a period characterised by heightened metabolic plasticity [25]. Ideally, these studies would integrate dietary assessment, microbiome sequencing and cardiometabolic biomarker profiling in order to characterise the dynamic interactions between diet, microbial metabolism and host endocrine responses during critical developmental periods [355]. Randomised controlled trials evaluating the efficacy, safety, and optimal dosing strategies of precision fermentation-derived prebiotics, postbiotics, and bioactive peptides in adolescent populations are essential [356,357]. These trials should also incorporate clinically relevant endpoints such as insulin sensitivity, endothelial function, inflammatory markers, and early cardiovascular risk indicators like arterial stiffness and altered lipid profiles.

In parallel, school- and population-level interventions are an important means of implementing microbiome-informed nutritional strategies [358,359]. Integrating functional foods and precision fermentation-derived ingredients into school nutrition programmes could substantially impact public health, especially when combined with policies that promote physical activity and restrict ultra-processed food consumption. School-based interventions are particularly effective as they reach large numbers of adolescents and encourage the development of healthy dietary behaviours in the long term during critical stages of development [360]. Meanwhile, advances in multi-omics technologies—including metagenomics, metabolomics and endocrine profiling—will enable the development of more precise, personalised nutritional strategies, tailored to individual microbiome and hormonal phenotypes [359,360]. Integrating artificial intelligence and systems biology approaches could enhance our ability to interpret complex multi-omics datasets, helping us to identify microbiome-derived biomarkers and personalised dietary targets for reducing cardiometabolic risk.

From a sustainability perspective, future research should focus on the environmental implications of scaling up precision fermentation technologies. Robust life-cycle assessments are particularly needed to quantify their environmental footprint and evaluate their potential to reduce dependence on resource-intensive, animal-based food systems [352]. Scaling up precision fermentation could contribute to more resilient, environmentally sustainable food systems by decoupling the production of functional nutrients from traditional agricultural constraints. Policymakers and educators will play a crucial role in this context, ensuring equitable access to precision fermentation-based innovations and integrating them into SDG-aligned frameworks that support planetary health and adolescent cardiometabolic resilience. Ultimately, successfully integrating precision fermentation into public health nutrition will require coordinated action across the scientific, regulatory and educational sectors to translate emerging biotechnological solutions into practical, accessible and socially equitable strategies for improving adolescent health.

## 11. Conclusions

Adolescence is a critical period of biological sensitivity and developmental dynamism, during which dietary exposure interacts closely with the gut microbiome and endocrine system to shape long-term cardiovascular risk trajectories. This review synthesises evidence that highlights how early cardiometabolic risk arises from the interplay between biological vulnerability, lifestyle behaviours, and environmental factors. Notably, the diet–microbiome–hormone axis emerges as a pivotal framework for early metabolic programming. This axis involves critical pathways such as SCFA production, bile acid metabolism, inflammatory signalling and endocrine regulation, which is mediated by the GH/IGF-1 axis, leptin, insulin and sex steroids.

The growing prevalence of obesity, dyslipidaemia, hypertension and insulin resistance among Polish adolescents reflects global trends and highlights the need for early preventive strategies. Diets characterised by a high intake of ultra-processed foods and low physical activity contribute to dysbiosis, metabolic endotoxemia and endothelial dysfunction, thereby accelerating cardiovascular risk from a young age. Conversely, dietary patterns rich in fermentable substrates support microbial diversity, enhance SCFA production, and promote metabolic resilience. However, conventional dietary approaches alone may not address the complexity of the microbiome-host-endocrine interaction network during adolescence.

In this context, precision fermentation is a promising, innovative biotechnological strategy for targeted nutritional intervention. By enabling the scalable production of well-defined bioactive compounds, including prebiotic oligosaccharides, postbiotics, bioactive peptides, and microbial lipids, precision fermentation has the potential to modulate key metabolic and endocrine pathways involved in cardiometabolic health. These compounds can improve the composition of the gut microbiota, stimulate SCFA production, enhance insulin sensitivity, reduce inflammation and support endothelial function. This provides a grounded approach to the early prevention of cardiovascular disease.

Notably, integrating precision fermentation into adolescent nutrition is consistent with broader sustainability objectives, such as reducing environmental impact and improving the efficiency of food production systems. Combined with public health strategies, such as improving school nutrition environments, limiting ultra-processed food consumption, and promoting physical activity, precision fermentation can contribute to a comprehensive, population-level approach to reducing cardiovascular risk.

Despite its potential, further longitudinal and well-designed clinical studies are needed to confirm the effectiveness, safety and long-term metabolic impact of precision fermentation-derived ingredients on adolescents. Future efforts should prioritise interdisciplinary collaboration between nutrition science, microbiome research, biotechnology and public health policy, ensuring these innovations can be translated into practical, accessible and equitable solutions.

## Figures and Tables

**Table 1:** In vitro and in vivo studies examining the interactions between diet, the microbiome and hormones, and their impact on early cardiovascular risk in adolescents and related experimental models.

Experimental Model or Study Population	Study Design and Methodological Approach	Experimental or Clinical Focus and Analytical Framework	Principal Findings and Pathways Leading to Observed Outcomes	References
Wistar albino rats on high-fat diet (HFD)	In vivo (animal)	Assessment of high-fat diet effects on cardiometabolic markers	HFD → ↑ lipids, ↑ insulin/leptin/glucose, ↑ cardiac oxidative stress	[144]
Adolescents (Nurses’ Health Study II)	Cohort (human)	Evaluation of adolescent diet quality and adult CVD risk	Higher diet quality in adolescence → ↓ hypertension, ↓ dyslipidemia in adulthood	[16]
Adolescents from EU countries	Clinical (observational)	Serum TMAO and inflammatory markers	↑ TMAO → ↑ BMI, ↑ CRP	[138]
Children with obesity	Clinical (cross-sectional)	TMAO associations with vascular function and metabolism	↑ TMAO → ↓ vascular elasticity	[145]
Polish adolescents	Epidemiological	Dietary patterns and CVD indicators	Low fiber/vegetable intake → ↑ LDL, ↑ blood pressure	[17]
ApoE^−/−^ mice on choline-rich diet	In vivo (transgenic)	TMAO effects on atherosclerosis	↑ TMAO → ↑ plaque formation, ↑ vascular inflammation	[146]
HFD-fed rats during Ramadan fasting	In vivo (animal)	Effects of intermittent fasting on lipid profile	Intermittent fasting → ↓ body weight, ↓ lipids	[147]
Children and adolescents	Literature review	Plant-based diets and CVD risk factors	Plant-based diet → improved lipid profile, better metabolic markers	[148]
Adults with CVD or CVD risk	Randomized controlled trials	Vegetarian diet effects on CVD markers	Vegetarian diet (6 months) → ↓ LDL-C, ↓ HbA1c, ↓ body weight	[149]
SHIME gut fermentation model	In vitro	Fiber fermentation and SCFA production	Fiber → ↑ butyrate, ↑ propionate	[150]
Human endothelial cells (HUVEC)	In vitro	TMAO effects on pro-inflammatory gene expression	TMAO → NF-κB activation → ↑ leukocyte adhesion	[142]
Endothelial barrier model in a flow-controlled bioreactor	In vitro (mechanical)	Vascular permeability under oxidative stress	TMAO exposure → endothelial barrier disruption	[151]
Brazilian adolescents (12–17 years)	Epidemiological (cross-sectional)	TMAO and cardiometabolic markers	↑ TMAO → ↑ BMI Z-score, ↑ waist circumference, ↑ CRP	[138]
Prospective cohort meta-analysis	Systematic review and meta-analysis	Microbiome metabolites (TMAO, L-carnitine, choline) and MACE risk	↑ TMAO/precursors → ↑ MACE, ↑ mortality	[152]
Mice on high-fat/high-sugar diet	In vivo	NLRP3 activation and cardiac function	HFD/HSD → NLRP3 activation → ↑ inflammation, ↑ cardiac injury; inhibition → protective	[153]
C57BL/6 mice on high-fat diet	In vivo	Cardiac fatty acid composition and vascular function	HFD → altered cardiac FA composition → impaired aortic flow → ↑ CVD risk	[154]

Abbreviations: HFD—high-fat diet; HSD—high-sugar diet; CVD—cardiovascular disease; TMAO—trimethylamine-N-oxide; SCFA—short-chain fatty acids; LDL—low-density lipoprotein; LDL-C—low-density lipoprotein cholesterol; HbA_1_c—glycated hemoglobin; CRP—C-reactive protein; BMI—body mass index; MACE—major adverse cardiovascular events; ApoE^−/−^—apolipoprotein E knockout; NF-κB—nuclear factor kappa-light-chain-enhancer of activated B cells; HUVEC—human umbilical vein endothelial cells; FA—fatty acids; SHIME—Simulator of the Human Intestinal Microbial Ecosystem; EU—European Union. Symbols: ↑ increase/elevation; ↓ decrease/reduction; → indicates an association, pathway, consequence, progression, or causal relationship between variables.

## Data Availability

No new data were created or analysed in this study.

## Abbreviations

ACE—angiotensin-converting enzyme; ApoE^−/−^—apolipoprotein E knockout; BCAA—branched-chain amino acids; BMI—body mass index; CRP—C-reactive protein; CVD—cardiovascular disease; DHA—docosahexaenoic acid; ECC—enteroendocrine cells; EPA—eicosapentaenoic acid; FA—fatty acids; GH—growth hormone; GHS-R—growth hormone secretagogue receptor; GLP-1—glucagon-like peptide-1; GOS—galacto-oligosaccharides; GPR41/43—G-protein–coupled receptor 41/43; HbA_1_c—glycated hemoglobin; HFD—high-fat diet; HSD—high-sugar diet; HUVEC—human umbilical vein endothelial cells; IPP—isoleucine-proline-proline; LDL—low-density lipoprotein; LDL-C—low-density lipoprotein cholesterol; LPS—lipopolysaccharide; MACE—major adverse cardiovascular events; MyD88—myeloid differentiation primary response 88; NAFLD—non-alcoholic fatty liver disease; NF-κB—nuclear factor kappa-light-chain-enhancer of activated B cells; NO—nitric oxide; NOD1—nucleotide-binding oligomerization domain-containing protein 1; PYY—peptide YY; RCT—randomized controlled trial; SCFAs—short-chain fatty acids; SHIME—Simulator of the Human Intestinal Microbial Ecosystem; TG—triglycerides; TLR4—toll-like receptor 4; TMAO—trimethylamine-N-oxide; VLDL—very-low-density lipoprotein; VPP—valine-proline-proline.
